# Investigating the impact of a pharmacist intervention on inappropriate prescribing practices at hospital admission and discharge in older patients: a secondary outcome analysis from a randomized controlled trial

**DOI:** 10.1177/20420986241299683

**Published:** 2024-11-15

**Authors:** Beate Hennie Garcia, Katharina Kaino Omma, Lars Småbrekke, Jeanette Schultz Johansen, Frode Skjold, Kjell Hermann Halvorsen

**Affiliations:** Department of Pharmacy, Faculty of Health Sciences, UiT the Arctic University of Norway, Tromsø, Tromsoe, 9037, Norway; Hospital Pharmacy of North Norway Trust, Tromsø, Norway; Department of Pharmacy, Faculty of Health Sciences, UiT the Arctic University of Norway, Tromsø, Norway; Department of Pharmacy, Faculty of Health Sciences, UiT the Arctic University of Norway, Tromsø, Norway; Hospital Pharmacy of North Norway Trust, Tromsø, Norway; Department of Pharmacy, Faculty of Health Sciences, UiT the Arctic University of Norway, Tromsø, Norway; Department of Pharmacy, Faculty of Health Sciences, UiT the Arctic University of Norway, Tromsø, Norway

**Keywords:** pharmacist intervention, potential inappropriate medication (PIM), potential prescribing omission (PPO), Screening Tool to Alert to Right Treatmen (START), Screening Tool of Older People’s Prescriptions (STOPP)

## Abstract

**Background:**

Inappropriate medication prescribing in older patients increases the risk of poorer health outcomes and increased costs. The IMMENSE trial, integrated a clinical pharmacist into the health care team, to improve medication therapy among older patients, and to investigate the impact on acute revisits to hospital.

**Objectives:**

This study investigated the prevalence of potentially inappropriate medications (PIMs) and prescribing omissions (PPOs) at hospital admission and discharge. It also explored the impact of the pharmacist intervention on PIMs and PPOs, and other factors associated with PIMs and PPOs at discharge.

**Design:**

The STOPP/START criteria version 2 were retrospectively applied at admission and discharge. PIM and PPO changes were compared, and Poisson regression was used to assess factors influencing prevalence at discharge.

**Results:**

At hospital admission, PIM prevalence was 58.6% among intervention patients and 64.8% among control patients. PPO prevalence was 55.3% and 55.5%, respectively. A larger proportion of PIMs identified at admission were resolved by discharge in the intervention group (42.9%) compared to the control group (27.4%). No difference was seen for PPOs. Poisson regression identified a significantly higher risk for PIMs at discharge in the control group compared to the intervention group (IRR 1.255; 95% CI 1.063–1.480, *p* = 0.007), but no effect for PPOs. Patients living in a nursing home, a home care facility, or an institution showed a higher risk of PPOs at discharge compared to patients living at home (IRR 1.378; 95% CI 1.156–1.644, *p* < 0.001).

**Conclusion:**

The IMMENSE intervention significantly reduced the risk of PIMs at discharge, with no effect on PPOs. Living in nursing homes, home care facilities, or institutions prior to hospitalization increased the risk of PPOs at discharge. Pharmacists may contribute to improved medication appropriateness in older hospitalized patients.

## Plain language summary

How a pharmacist’s contribution in a hospital ward can reduce inappropriate medication prescribing in older patients: insights from a study

**Why Was This Study Done?** The IMMENSE trial explored whether integrating a clinical pharmacist into the healthcare team could reduce inappropriate medications and improve health outcomes for older patients, potentially decreasing unnecessary hospital revisits.

**What Did the Researchers Do?** The researchers assessed the presence of potentially inappropriate medications (PIMs) and prescribing omissions (PPOs) at hospital admission and discharge. They used the STOPP/START criteria to evaluate medication lists to compare changes and understand factors influencing these issues at discharge.

**What Did the Researchers Find?** The study revealed that 57-65% of patients had inappropriate medications, and 55-57% experienced prescribing omissions during their hospital stay. Patients in the group with a pharmacist on their healthcare team had a significant reduction in PIMs at discharge. However, there was no significant change in PPOs. Additionally, patients from nursing homes or similar facilities were more likely to have PPOs at discharge compared to those living at home.

**What Do the Findings Mean?** The findings suggest that including a pharmacist in the healthcare team significantly helps in reducing inappropriate medications among hospitalized older patients, enhancing medication safety. However, the intervention did not significantly affect prescribing omissions. This indicates a need for targeted strategies to address omissions, especially for patients in nursing homes or care facilities. The role of pharmacists is crucial in improving medication management and safety in hospital settings, particularly for the elderly.

## Introduction

Inappropriate medication prescribing is harmful, especially for older patients who are at increased risk.^1^ Potentially inappropriate prescribing (PIP) encompasses both potentially inappropriate medications (PIMs), mis-prescribing (e.g., an inappropriate frequency, dose, or duration), or the failure to prescribe medications that would likely benefit patients; potential prescribing omissions (PPOs).^2,3^ PIPs are highly prevalent in older adults,^4,5^ and are associated with adverse drug events (ADEs), increased healthcare use, elevated costs, and reduced health-related quality of life.^6–8^ Recent studies show a prevalence of PIPs in older patients up to 97%, with variations across regions and economic statuses.^9–12^ Additionally, nearly half of hospitalized older patients experience PIPs.^13^

To assess medication use in relation to PIPs, several criterion-based tools have been developed, the most well-known being Screening Tool of Older People’s Prescriptions (STOPP) and Screening Tool to Alert to Right Treatment (START).^14^ STOPP addresses issues such as duplicate prescriptions and drug interactions (PIMs), while START focuses on medications that should be prescribed in certain conditions (PPOs).^15^ STOPP application has linked PIMs to increased adverse reactions and hospital admissions,^8,16^ while clinical application of the STOPP/START criteria has been shown to reduce ADEs and medication costs for acutely ill older patients.^17^ STOPP/START version 2 was published in 2015,^5^ while version 3 was published in 2023.^18,19^ The STOPP/START criteria have been converted into software algorithms and integrated into a clinical decision support system to facilitate their use in clinical practice.^20^

Several clinical intervention studies utilizing STOPP/START criteria to enhance medication appropriateness focus on outcomes such as ADEs, mortality, rehospitalizations, or length of hospital stay, with few reporting the actual reduction in PIMs and PPOs on the individual patient level.^10,21,22^ From the creators of the STOPP/START criteria, two RCTs showed that implementing STOPP/START version 2 recommendations can reduce in-hospital adverse drug reactions (ADRs) in older adults^3^ although detailed data on START/STOPP recommendations were provided in only one of these studies, and not at the individual patient level.^17,23^ The OPERAM study, a cluster-randomized trial across four European countries, reported changes in medication overuse and underuse based on STOPP/START version 22.^21^ The intervention involved structured pharmacotherapy optimization supported by an algorithm-based tool aimed at reducing inappropriate prescribing. A mean of 2.8 recommendations were identified per patient, with a mean of 1.2 implemented after 2 months. An OPERAM substudy reported a 39% acceptance rate of software-generated signals based on STOPP/START criteria, suggesting a reduction in PIPs during hospitalization.^20^ The OPTICA trial reported that 28% of STOPP and 14% of START recommendations from a similar PIP identifying software algorithm were implemented.^24,25^

Clinical pharmacists play a crucial role in improving prescribing appropriateness and preventing drug-related problems, particularly among vulnerable populations such as the elderly.^26^ Their responsibilities extend beyond dispensing medications to actively conduct pharmaceutical care planning and participating in patient care teams. By conducting comprehensive medication reviews and assessments, clinical pharmacists identify both PIMs and PPOs.^27^ They provide recommendations on tailoring medication regimes based on individual patient needs, comorbidities, and potential drug interactions. This proactive involvement ensures optimized therapeutic outcomes and minimizes the risks of ADEs, significantly improving quality of prescribing and health-related quality of life of the patients.^28^

In Norway, two RCTs aiming to improve medication therapy in older adults have recently been conducted, the IMMENSE and the OPERA trial.^29,30^ Both trials investigated the impact of including clinical pharmacists in hospital teams on rehospitalizations, revisits, and all-cause mortality, but failed to show any impact. A substudy of the OPERA trial applied the STOPP/START criteria to investigate the effect of the intervention on the quality of drug treatment at discharge. A significant reduction in the mean number of PPOs from hospital admission was identified in the intervention group, but no impact with regard to PIMs was observed.^22^ In this study, we investigated the secondary endpoint of the IMMENSE trial *“Change in potentially inappropriate medications prescribed identified by STOPP/START version 2 from admission to discharge.*”^31^ This was done by investigating the prevalence of PIMs and PPOs at hospital admission and discharge, exploring the impact of the pharmacist intervention on PIMs and PPOs at discharge, as well as other factors associated with PIMs and PPOs at discharge.

## Methods

### Study design and reporting criteria

This study analyses data collected in the IMMENSE study, conducted from September 2016 to December 2019 at two internal medicine wards at the University Hospital of North Norway. Follow-up was performed for 12 months after hospitalization and ended on December 2020. For complete information about the study, we refer to the published protocol, the main study, and the fidelity study.^27,29,31^ This manuscript has been prepared applying the CONSORT guidelines for reporting parallel group randomized trials.^32^

### Study setting

The IMMENSE study took place in both a geriatric internal medicine ward and a general internal medicine ward at the University Hospital of North Norway. The geriatric ward specializes in treating older patients with complex acute medical conditions, staffed by physicians with expertise in geriatric medicine. The general medicine ward provides care for patients suffering from conditions such as stroke, pulmonary, kidney, and endocrine diseases, including those with geriatric issues. Prior to the study, pharmacists were not part of the staff in these hospital wards.

### Participants, recruitment, and randomization

The IMMENSE study included acutely admitted patients aged ⩾70 years who were willing to provide written informed consent, either themselves or via the next of kin. Exclusion criteria included being admitted to the study ward for over 72 h before eligibility assessment, transferring or discharging from other wards during the index stay, inability to understand Norwegian (patient or next of kin), terminal illness or short life expectancy, planned discharge on the day of inclusion, occupying a bed in a study ward but under non-study ward physician care, or requiring intervention from a study pharmacist (before randomization or in the control group). Study pharmacists screened and recruited eligible patients during their weekday work hours from 8:00 AM to 3:30 PM. To minimize selection bias, patients were approached for inclusion in a predetermined sequence. Following the collection of baseline data, patients were randomized by the study pharmacists in a 1:1 relationship through a web-based service. The randomization involved permuted block sizes that were both unknown and varied in size.

### Sample size calculation

The sample size for the primary outcome in the IMMENSE trial was determined based on a study by Gillespie et al., which utilized the same composite endpoint of a 30-day hospital revisit.^26^ The Gillespie study evaluated the effectiveness of a multifaceted intervention, including post-discharge activities conducted by ward-based pharmacists, in reducing morbidity and hospital visits among patients aged 80 years and older. This study reported a 16% reduction in all-cause hospital visits in the intervention group. Anticipating an annual rate of 1.7 acute hospital admissions and Emergency Department (ED) visits within our patient cohort, and aiming for a 5% significance level with 80% power, the IMMENSE trial planned to enroll 250 patients per group, taking potential dropouts into account.

### The intervention

The intervention concerned including clinical pharmacists in the interdisciplinary team at the two hospital wards caring for older patients, aiming to improve medication appropriateness and medication safety. The pharmacists worked according to the Integrated Medication Management (IMM) model^34,35^ which comprises four steps; (i) medication reconciliation at admission, (ii) medication review as needed during hospitalization, (iii) medication counseling to patient/next of kin, and (iv) compilation of an accurate and complete medication list for transfer to the next care level. A step (v) was added, comprising a phone call to the subsequent healthcare level to discuss any changes in medication regimes or drug-related issues. In addition, interdisciplinary team discussions were carried out during daily meetings and whenever medication-related problems were identified. During medication reviews, the clinical pharmacists consulted tools for prescribing optimization as needed, including the STOPP/START criteria.^5^ Control group patients received standard care from the same ward team, except for the services provided by the pharmacist. Medication reconciliation was performed by physicians, who were also responsible for medication reviews, discharge summaries, patient counseling, and communication with primary care.

### Scoring tools

We retrospectively identified PIMs and PPOs at hospital admission and discharge in all patients using the Norwegian translation of the STOPP/START version 2 criteria.^5^ Information sources applied included (i) the medication list at admission (before medication reconciliation to treat both groups similarly), (ii) medication list at discharge identified in the discharge summary, (iii) laboratory values and test results, and (iv) relevant clinical measures during hospitalization (e.g., blood pressure and heart rate). No information in the pharmacist notes (handwritten or in medical records) nor the physician’s assessment of medical therapy in discharge notes was applied. We excluded 15 STOPP criteria (A1, A2, B5, B6, B9, B10, C8, C9, D2, D9, D12, F3, G4, H3, and L1) and 11 START criteria (A2, A4, B3, C2, C5, D1, E1, G3, H1, I1, and I2) because of missing information in our dataset on contraindications, laboratory values, degree of disease and medication history, and due to the retrospective application. Consequently, 89 criteria were applied per medication list in and out of hospital for all patients, that is, 85440 criterion applications. See Supplemental 1 for criteria and application guide.

### Application of the scoring tools

At hospital admission, we applied patients’ medication lists denoted in the hospital admission journal. This was available *before* randomization of patients, and before the clinical pharmacist intervention started. At hospital discharge, we used the medication list in the hospital discharge summary. Although the discharge summary was written by the physician for both study groups, the medication lists were compiled by the intervention pharmacists and copy-pasted into the discharge summary by the physician.

The rater applying the scoring tools (KKO) was blinded for group allocation. Each criterium was identified on the patient level. Two drug groups gave rise to scoring for more than one criterium, that is, benzodiazepine use for more than 4 weeks would give a score in both STOPP-D5 and STOPP-K1, while regular use of opioids without laxantia would give a score to both STOPP-L2 and START-H2. We kept both scores in the analyses.

### Outcome of scoring categories

For the presence of PIMs and PPOs identified in patients at admission and discharge, we defined four categories 0-0: neither present at admission nor discharge, 1-0: present at admission but not at discharge, 0-1: not present at admission but at discharge, and 1-1: present at admission and at discharge.

### Statistics

For data management and analyses we applied Microsoft^®^ Excel for Mac version 16.69.1, and IBM^®^ SPSS Statistics for Mac version 29.0.0.0 (241). Results are expressed with means, proportions, standard deviations (SDs), medians, interquartile range (IQR), minimum and maximum values, and 95% confidence intervals (CIs) where appropriate.

To investigate intra-rater agreement, the main rater (KKO) randomly selected medication lists at hospital discharge for 15 patients and performed a new scoring 4 weeks after finalizing scoring for the whole patient population. To investigate inter-rater agreement, a second rater (JSJ) also performed scoring of the medication lists at hospital discharge for the same 15 patients. We calculated intra- and inter-rater agreement of the scoring by applying Cohen’s kappa (κ) statistics.

To investigate differences between the study groups and factors associated with PIMs and PPOs at discharge, we developed two Poisson regression models. Poisson regression was chosen as PIM/PPO outcome variables followed by a Poisson distribution. In both models, we applied the number of PIMs and PPOs as the outcome variable, respectively, and adjusted for the following: number of PIM/PPOs at hospital admission, group allocation (intervention or control group), sex, age, number of medications at hospital admission, Charlson Comorbidity Index,^37,38^ living status (home or, institution, i.e., nursing home or home care facility). Excluding any of the factors based on collinearity did not notably alter model parameters, that is, Pearson’s chi-square or Akaike information criterion (AIC) values. Consequently, we kept the variables in the model. See Supplemental 2 for a directed acyclic graph describing relationship between the outcome and potential associated variables in the model selection developed by the free software at www.daggity.net. The significance level in all analyses was set to *p* < 0.05.

### Ethics

This research was performed in accordance with ethical guidelines stated by the Helsinki Declaration. The Norwegian Centre for Research Data and the Norwegian Data Protection Authority granted ethical approval (Project number 41366). Participants in the IMMENSE study have given informed written consent for data collection, analysis, and storage.

## Results

### The patient population

The IMMENSE trial included 480 patients: 244 randomized to the intervention group and 236 to the control group. The two groups were similar regarding age, sex, and comorbidity. The control group used more medications at hospital admission and discharge compared to the intervention group, see Table 1.^29^

**Table 1.** General characteristics of the IMMENSE study population (*n* = 480).^19^

Characteristics	Intervention group (*n* = 244)	Control group (*n* = 236)
Age in years, mean (SD)	83.3 (6.4)	83.0 (6.3)
Female sex, *n* (%)	152 (62.3)	127 (53.8)
Number of medications at hospital admission, Median (IQR)		
Regular use	6 (4–9)	7 (4–10)
Use as needed “pro-re-nata”	2 (0–3)	2 (0–3)
Number of medications at hospital discharge, Median (IQR)		
Regular use	6 (4–9)	7 (5–10)
Use as needed “pro-re-nata”	2 (1–3)	2 (1–4)
Comorbidity, median score CCI (IQR)	2 (1–3.75)	2 (1–4)
Most frequent diagnoses at hospital admission, *n* (%)		
Hypertension	125 (51.2)	113 (47.9)
Atrial fibrillation	67 (27.5)	65 (27.5)
Asthma and chronic obstructive pulmonary disease	55 (22.5)	53 (22.5)
Diabetes Mellitus	50 (20.5)	52 (22.0)
Congestive Heart failure	40 (16.4)	36 (15.3)
Renal insufficiency	34 (13.9)	34 (14.4)
Dementia or chronic cognitive deficit	34 (13.9)	32 (13.6)
Living status before admission, *n* (%)		
Home	149 (61.1)	131 (55.5)
Not home*	95 (38.9)	105 (44.5)

* Nursing home, rehabilitation, or alternative residential care facilities.

CCI, Charlson comorbidity index; IQR, interquartile range; SD, Standard deviation.

### PIM and PPO prevalence

At hospital admission, we identified 289 PIMs and 242 PPOs in the intervention group, and 332 PIMs and 256 PPOs in the control group. PIM prevalence was 58.6% among intervention patients and 64.8% among control patients. Similarly, PPO prevalence was 55.3% among intervention patients and 55.5% among control patients. The median of PIMs and PPOs in both groups was 1, see Table 2.

**Table 2.** Overview of PIM and PPO prevalence at hospital admission and discharge in the intervention and control group of the IMMENSE population.

PIMs and PPOs	Intervention group (*n* = 244)		Control group (*n* = 236)	
	*n* (Prevalence)	Median (IQR)	*n* (Prevalence)	Median (IQR)
PIMs				
Admission	289 (58.6)	1 (0–6)	332 (64.8)	1 (0–6)
Discharge	252 (57.0)	1 (0–6)	336 (64.8)	1 (0–6)
PPOs				
Admission	242 (55.3)	1 (0–5)	256 (55.5)	1 (0–7)
Discharge	263 (55.8)	1 (0–6)	267 (56.8)	1 (0–9)

IQR, interquartile range; n, number of criterion scorings; PIM, potentially inappropriate medication; PPO, potential prescribing omission.

At hospital discharge, we identified 252 PIMs and 263 PPOs in the intervention group, and 336 PIMs and 267 PPOs in the control group. PIM prevalence was 57.0% among intervention patients and 64.8% among control patients. Similarly, PPO prevalence was 55.8% among intervention patients and 56.8% among control patients. The median of PIMs and PPOs in both groups was 1, see Table 2.

**Figure 1.**
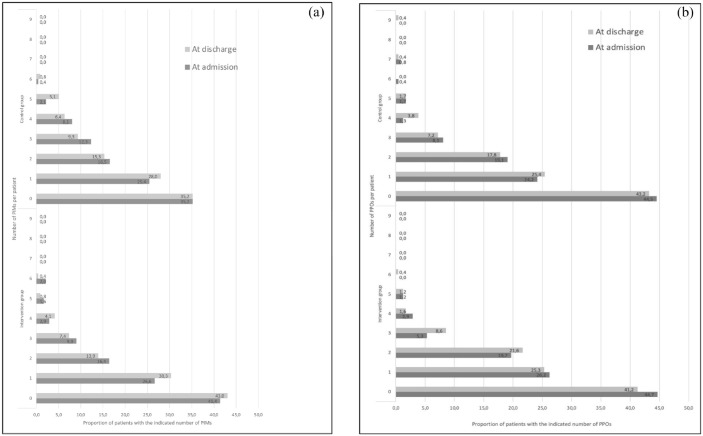
Distribution of PIMs (Panel a) and PPOs (Panel b) in the IMMENSE population at hospital admission and discharge. PIM, potentially inappropriate medications; PPOs, potentially prescribing omissions.

See Figure 1(a) and (b) for the distribution of PIMs and PPOs in the study groups. The number of PIMs identified per patient varied from 0 to 6 in both groups, as shown in Figure 1(a). In contrast, the number of PPOs per patient ranged from 0 to 6 in the intervention group and 0 to 9 in the control group, as depicted in Figure 1(b). Notably, for the one patient with nine PPOs identified at discharge in the control group, it is suspected that most medications were discontinued during the hospital stay without adequate documentation in the discharge summary. According to Figure 1(a), the proportion of patients with 0 PIMs increased at discharge in the intervention group (shown in the lower graph) but remained unchanged in the control group (shown in the upper graph). Figure 1(b) indicates a decrease in the proportion of patients with 0 PPOs from admission to discharge in both groups.

Among the 65 STOPP criteria applied, 47 (72%) gave rise to a PIM score, see Supplemental 3, Table 1. Eleven criteria gave rise to more than 75% of the scoring both at hospital admission and discharge, see Table 3. Use of z-hypnotics (criteria K4) increased during hospitalization in both groups, while benzodiazepines seemed to be reduced in the intervention group but increased in the control group. Among the 23 START criteria applied, 20 (87%) gave rise to a PPO score, see Supplemental 3, Table 2. Nine criteria gave rise to more than 75% of the scoring both at hospital admission and discharge, see Table 3. No differences were observed between the two groups.

**Table 3.** STOPP and START criteria giving rise to cumulative 75% of all scorings both at hospital admission and discharge in the total IMMENSE population (*n* = 480).

Criterion number	Criterion description	At hospital admission				At discharge			
		*n*	Patients with score (%)	% of crit	Cum % of scorings	*n*	Patients with score (%)	% of crit	Cum % of scorings
STOPP-K4	Hypnotic Z-drugs, for example, zopiclone, zolpidem, zaleplon (may cause protracted daytime sedation, ataxia)	100	20.8	16.1	16.1	115	24.0	19.6	19.6
STOPP-K1	Benzodiazepines (sedative, may cause reduced sensorium, impair balance)	85	17.7	13.7	29.8	86	17.9	14.6	34.2
STOPP-F2	PPI for uncomplicated peptic ulcer disease or erosive peptic esophagitis at full therapeutic dosage for >8 weeks (dose reduction or earlier discontinuation indicated)	46	9.6	7.4	37.2	38	7.9	6.5	40.6
STOPP-B7	Loop diuretic for dependent ankle edema without clinical, biochemical evidence, or radiological evidence of heart failure, liver failure, nephrotic syndrome, or renal failure (leg elevation and/or compression hosiery usually more appropriate)	40	8.3	6.4	43.6	23	4.8	3.9	44.6
STOPP-L2	Use of regular (as distinct from PRN) opioids without concomitant laxative (risk of severe constipation)	39	8.1	6.3	49.9	25	5.2	4.3	48.8
STOPP-K2	Neuroleptic drugs (may cause gait dyspraxia, Parkinsonism)	31	6.5	5.0	59.9	37	7.7	6.3	61.2
STOPP-A3	Any duplicate drug class prescription (e.g., two concurrent NSAIDs, SSRIs, loop diuretics, ACE inhibitors, anticoagulants)	31	6.5	5.0	59.9	36	7.5	6.1	61.2
STOPP-K3	Vasodilator drugs (e.g., alpha-1 receptor blockers, calcium channel blockers, long-acting nitrates, ACE inhibitors, angiotensin II receptor blockers) with persistent postural hypotension, that is, recurrent drop in systolic blood pressure ⩾20 mmHg (risk of syncope, falls)	28	5.8	4.5	64.4	26	5.4	4.4	65.6
STOPP-L3	Long-acting opioids without short-acting opioids for break-through pain (risk of persistence of severe pain)	26	5.4	4.2	68.6	25	5.2	4.3	69.9
STOPP-D5	Benzodiazepines for ⩾4 weeks (no indication for longer treatment; risk of prolonged sedation, confusion, impaired balance, falls, road traffic accidents; all benzodiazepines should be withdrawn gradually if taken for more than 4 weeks as there is a risk of causing a benzodiazepine withdrawal syndrome if stopped abruptly)	24	5.0	3.9	72.5	22	4.6	3.7	73.6
STOPP-M1	Concomitant use of two or more drugs with antimuscarinic/anticholinergic properties (e.g., bladder antispasmodics, intestinal antispasmodics, tricyclic antidepressants, first-generation antihistamines) (risk of increased antimuscarinic/anticholinergic toxicity)	17	3.5	2.7	75.2	18	3.8	3.1	76.7
START-E4	Bone anti-resorptive or anabolic therapy (e.g., bisphosphonate, strontium ranelate, teriparatide, denosumab) in patients with documented osteoporosis, where no pharmacological or clinical status contraindication exists (Bone Mineral Density T-scores -> 2.5 in multiple sites) and/or previous history of fragility fracture(s)	104	21.7	20.9	20.9	103	21.5	19.4	19.4
START-E3	Vitamin D and calcium supplements in patients with known osteoporosis and/or previous fragility fracture(s) and/or Bone Mineral Density T-scores more than −2.5 in multiple sites	81	16.9	16.3	37.1	81	16.9	15.3	34.7
START-A6	ACE inhibitor with systolic heart failure and/or documented coronary artery disease	38	7.9	7.6	44.8	46	9.6	8.7	43.4
START-B1	Regular inhaled β2 agonist or antimuscarinic bronchodilator (e.g., ipratropium, tiotropium) for mild-to-moderate asthma or COPD	27	5.6	5.4	50.2	28	5.8	5.3	48.7
START-A7	Beta-blocker with ischemic heart disease	27	5.6	5.4	55.6	33	6.9	6.2	54.9
START-A5	Statin therapy with a documented history of coronary, cerebral, or peripheral vascular disease, unless the patient’s status is end-of-life or age is > 85 years	31	6.5	6.2	69.7	33	6.9	6.2	65.8
START-H2	Laxatives in patients receiving opioids regularly	39	8.1	7.8	69.7	25	5.2	4.7	65.8
START-E2	Bisphosphonates and vitamin D and calcium in patients taking long-term systemic corticosteroid therapy	32	6.7	6.4	76.1	28	5.8	5.3	71.1
START-C3	Acetylcholinesterase inhibitor (e.g., donepezil, rivastigmine, galantamine) for mild-moderate Alzheimer’s dementia or Lewy Body dementia (rivastigmine)	16	3.3	3.2	79.3	27	5.6	5.1	76.2

ACE, angiotensin converting Enzyme; COPD, chronic obstructive pulmonary disease; Crit., criterion; Cum., cumulative; NSAIDs, non-steroidal anti-inflammatory drugs; Pat., patient; PPI, proton pump inhibitor; PRN, pro-re-nata; SSRIs, selective serotonin receptor inhibitors.

### Change in PIMs and PPOs from admission to discharge

Examining PIMs and PPOs at hospital admission for individual patients, some were still present at discharge (category 1–1), some were resolved by discharge (category 1–0), and some appeared only at discharge but were not observed at admission (category 0–1), see Table 4. The percentage of PIMs identified at admission and resolved by discharge was higher in the intervention group (42.9%) compared to the control group (27.4%). Conversely, the percentage of PIMs present at both admission and discharge was lower in the intervention group (57.1%) than in the control group (72.6%). The proportions of PPOs resolved by discharge were similar between the intervention group (18.6%) and the control group (19.9%). Likewise, the proportions of PPOs present at both admission and discharge were nearly the same in the intervention group (81.4%) and the control group (80.1%). The number of PIMs and PPOs identified only at discharge was also comparable between the intervention and control groups. The STOPP criterion most frequently giving rise to PIMs at discharge that was not observed at admission was K4 *“Hypnotic Z*-drugs, for example, zopiclone, zolpidem, zaleplon. The START criteria most frequently giving rise to new PPOs at discharge, not observed at admission, were D2 *“Fibre supplements for diverticulosis with a history of constipation*” and C3 “*Acetylcholinesterase inhibitor for mild-moderate Alzheimer’s dementia or Lewy Body dementia.*”

**Table 4.** Change in PIM and PPO score in the individual patient from hospital admission to discharge in the IMMENSE population (*n* = 480).

Outcome categories*	PIMs				PPOs			
	Intervention group (*n* = 244)		Control group (*n* = 236)		Intervention group (*n* = 244)		Control group (*n* = 236)	
	*n*	(%)**	*n*	(%)**	*n*	(%)**	*n*	(%)**
1-0	124	42.9	91	27.4	45	18.6	51	19.9
1-1	165	57.1	241	72.6	197	81.4	205	80.1
0-1	87	–	95	–	66	–	62	–
0-0	15484	–	14913	–	5304	–	5110	–

* 1-0: Score at hospital admission, no score at hospital discharge, 1-1: Score both at hospital admission and at hospital discharge, 0-1: No score at hospital admission, but score at hospital discharge, 0-0 no score neither at hospital admission nor at discharge.

** percent of the number identified at hospital admission.

PIM, potential inappropriate medication; PPO, potential prescribing omission.

### The impact of the pharmacist intervention and factors associated with PIMs and PPOs at discharge

The regression model for PIMs showed that patients in the control group had a higher risk for PIMs at discharge than patients in the intervention group, with an IRR of 1.255 (95% CI 1.063–1.480, *p* = 0.007), see Table 5. The regression model for PPOs showed no effect of the intervention, with an IRR of 0.903 (95% CI 0.757–1.078). The PPO regression model showed that patients not living at home (i.e., in nursing homes or home care facilities) prior to hospital admission had a higher risk of PPOs at discharge than patients living at home, with an IRR of 1.378 (95% CI 1.156–1.644, *p* < 0.001).

**Table 5.** Factors associated with risk of PIMs (a) and PPOs (b) at hospital discharge in the IMMENSE population (*n* = 480).

Factors associated with risk for	**β**-value	Std. Error	IRR	95% CI		*p*
**(a) PIMs defined by the STOPP criteria at discharge, Pearson’s chi^2^ = 0.886**						
intercept	−1.394	0.5846	0.248	0.079	0.780	0.017
PIMs at admission, n	0.398	0.0304	1.489	1.403	1.580	<0.001*
Group^a^	0.227	0.0845	1.255	1.063	1.480	0.007*
Sex	−0.014	0.0872	0.986	0.831	1.169	0.868
Age	0.006	0.0070	1.006	0.992	1.020	0.392
CCI	0.004	0.0230	1.004	0.960	1.051	0.855
Number of medications	0.018	0.0100	1.018	0.999	1.039	0.068
Living status^b^	0.105	0.0849	1.111	0.941	1.312	0.215
**(b) PPOs defined by the START criteria at discharge, Pearson’s chi^2^ = 0.731**						
intercept	−1.215	0.5732	0.297	0.096	0.912	0.034
PPOs at admission, n	0.468	0.0249	1.597	1.521	1.677	<0.001*
Group^a^	−0.101	0.0902	0.903	0.757	1.078	0.260
Sex	−0.138	0.0914	0.872	0.729	1.043	0.133
Age	0.005	0.0069	1.005	0.992	1.019	0.456
CCI	0.013	0.0229	1.013	0.968	1.059	0.576
Number of medications	0.009	0.0087	1.009	0.992	1.027	0.288
Living status^b^	0.321	0.0898	1.378	1.156	1.644	<0.001*

a **Control group** versus intervention group

b **Not home** (in institution, nursing home, home care facility) versus home.

* significant with a p-value < 0.05.

CI, confidence interval; CCI, Charlson Comorbidity Index score; IRR, Incidence rate ratio; PIM, potential inappropriate medication; PPO, potential prescribing omissions.

### Reliability testing

According to McHugh,^36^ intra-rater agreement was interpreted as almost perfect (κ = 0.89), and inter-rater considerable (κ = 0.65). Most disagreements arose concerning three START criteria involving the use of acetylcholinesterase inhibitors for mild-to-moderate Alzheimer’s dementia or Lewy Body dementia (C3), the use of vitamin D and calcium supplements in patients with known osteoporosis and/or previous fragility fractures (E3), and use of bone anti-resorptive or anabolic therapy (e.g., bisphosphonate, strontium ranelate, teriparatide, and denosumab) in patients with documented osteoporosis where no pharmacological or clinical contraindications exist (E4). Consequently, we applied the original scoring in our data analyses.

## Discussion

This study evaluated the prevalence of PIMs and PPOs at hospital admission and discharge in the IMMENSE population, a randomized controlled study investigating the impact of pharmacist intervention to optimize medication use in older hospitalized patients. Additionally, the study investigated the impact of this pharmacist intervention on PIMs and PPOs, along with other factors influencing these outcomes at discharge. We observed a high prevalence of both PIMs and PPOs in the IMMENSE population, in both study groups at hospital admission as well as at discharge. After adjusting for the number of PIMs and PPOs at hospital admission, we identified a significantly increased risk of PIMs at hospital discharge in the control group compared to the intervention group, suggesting a beneficial effect of the IMMENSE intervention on PIMs. However, no significant impact of the intervention was observed on PPOs. We also identified an increased risk of PPOs at hospital discharge among patients residing in nursing homes, home care facilities, or other kind of institutions, highlighting a need for increased focus on this patient group.

The consistently high prevalence of PIM and PPOs aligns with previous research and findings.^9,39,40^ Moriarty et al.^39^ studied prevalence of PIMs and PPOs among community-dwelling older people in Ireland, applying different screening tools, among them START and STOPP.^39^ The prevalences of PIMs and PPOs were 52.7% and 38.2%, respectively, applying the STOPP and START criteria. In a literature review from 2023 investigating PIMs and PPOs among nursing home residents, Planelle et al. identified a PIM prevalence between 67.8% and 87.7% applying the STOPP criteria, and between 39.5% and 99.7% applying the START criteria.^40^ From the systematic review and meta-analysis conducted by Tian et al.,^9^ it seems like the overall prevalence of PIM use in outpatient services is a little bit lower. From 94 articles with 132 prevalence estimates, they identified a pooled PIM prevalence of 36.7% (95% CI 33.4%–40.0%) in nearly 371 million older patients from 17 countries worldwide. The authors emphasized that the prevalence is highest in low-income areas.

Many studies highlight the risk of ADEs and negative impacts on quality of life and life expectancy caused by PIMs and PPOs.^9,10,22^ In 2021, Mekonnen et al. conducted a systematic review and meta-analysis, investigating the association between PIP and health-related and system-related outcomes in hospitalized older adults. They found that PIP was associated with 91%, 60%, and 26% increased odds of ADE-related hospital admissions, functional decline, and ADRs & ADEs, respectively.^10^ To mitigate these risks, Santos et al. recommend a multifaceted approach, including promoting appropriate prescribing practices, enhancing interprofessional collaboration, integrating the expertise of pharmacists as medication specialists, implementing electronic prescribing support systems, and focusing on patient education for medication therapy adherence.^41^ While some countries have made progress in these areas, particularly within hospitals by, for example, implementing electronic medication records, prescriber support systems, and employing clinical pharmacists, few have developed and implemented a fully multifaceted approach. One major challenge worldwide is the access to updated patient medication lists shared across healthcare sectors, including pharmacies. Such a list, which is extremely valuable when prescribing, dispensing, and giving medications, is available only in few countries such as Sweden and Denmark. However, work is in progress, for example, in Norway where they are piloting a national collaboration for the “Patient’s medication list,” which should be available from different health care levels.^42^ In some countries like the USA, UK, Canada, and Australia, pharmacists have increasingly become integral members of interdisciplinary healthcare teams in various settings, including primary healthcare, ambulatory care, emergency departments, and hospital wards.^43–46^ Their roles have expanded to include independent management of pharmacotherapy clinics and active participation in patient care. In contrast, in Norway, the integration of pharmacists into healthcare teams has not yet achieved the level observed in these countries, which could potentially help mitigate the risk of PIMs and PPOs in the growing older population.

The observed higher risk for PIMs at discharge in the control group aligns with our expectations, given the many resolved medication discrepancies and medication-related problems for the IMMENSE intervention patients.^27^ This was not seen in the OPERA trial,^30^ but has been identified in other clinical pharmacist interventions.^47^ Consequently, incorporating clinical pharmacists into the interdisciplinary team to optimize medication use in older patients appears beneficial. Additionally, it may be economically beneficial, as suggested by Robinson et al. in the cost-utility analysis of the IMMENSE intervention, where the intervention dominated standard care for patients with a length of hospital stay shorter than 2 weeks.^48^ Although the IMMENSE trial did not demonstrate significant effects on acute revisits to the hospital, mortality, or time to rehospitalization,^29^ we have now observed a significant effect of the intervention on PIMs. The significant reduction of PIMs in the intervention group may potentially be linked to the higher health-related quality of life observed in the intervention group compared to the control group shortly after discharge.^49^ This higher health-related quality of life may have contributed to the beneficial cost-utility observed in some of the intervention patients.^48^ This relationship between PIMs and lower health-related quality of life has also previously been established in the literature.^50–52^

The lack of observed effect on PPOs in our study contrasts with findings from other studies such as OPERA, OPERAM, and the Irish studies.^17^, ^21–23^ Unlike the OPERA study conducted in a Norwegian hospital, which recruited multimorbid patients from an internal medicine ward without a specific focus on older adults,^30^ the IMMENSE study exclusively included older adults from a specialized geriatric department. These participants were predominantly frail, with advanced dementia and a high prevalence of inability to consent to study participation.^29^ Consequently, the intervention recommendationsa, particularly concerning therapy initiation and deprescribing, likely differed between the studies. This assertion is supported by the significant emphasis on deprescribing z-hypnotics and benzodiazepines in the IMMENSE study. These criteria represented more than 30% of all scorings at hospital admission, with an observed reduction in the intervention group compared to the control group.

Our findings suggest that living in a nursing home, institution, or community care facility increases the risk of having PPOs. This prompts reflection on prescribing practices and follow-up of medication therapy for older adults, particularly those nearing the end of life. One reason for under-prescribing in this frail population may be fear of causing ADEs,^53^ especially in polypharmacy patients.^54^ Also, deprescribing has achieved higher attention among older patients during the past two decades, promoting stopping rather than initiating medication therapy in older adults.^55^ Proactively applying the START criteria in clinical practice could help healthcare providers recognize under-prescribing and support informed decision-making involving patients and carers.

### Strengths and limitations

In this study, we used a randomized controlled design, which had a meticulous data collection with a complete dataset and high intra - and inter-rater validity. However, there are limitations. The accuracy of the medication lists at admission is uncertain, as they were compiled before patient enrollment and prior to thorough medication reconciliation. This could introduce bias when comparing medication status at admission and discharge, as the change identified may not be true based on incorrect medication lists. This should affect both groups equally, but may eventually affect the statistical estimates in either direction. Another concern pertains to the potential effect of hospital interventions on reducing PIMs and PPOs. The short hospital stays may not allow for significant medication changes, which could be more effectively managed in primary care with longer-term monitoring. This is supported by data from the fidelity study, describing a large proportion of therapy recommendations communicated to the next care level.^27^ If these were made before hospital discharge, we may have seen a larger effect on PIMs and PPOs. Furthermore, subtle changes like dosage adjustments or reductions in medications such as benzodiazepines may not be detected by the STOPP criteria, possibly leading to an underestimation of the intervention’s impact. With regards to generalizability, the patient population included in this study was comparable to older patients in other hospital settings, and the number of medication-related problems identified was very similar.^27^ Consequently, the study results may be applicable to other hospitals admitting older patients, where the pharmacists contribute with procedures related to the IMM methodology, that is, medication reconciliation, medication review, and communication of appropriate medication use to patients and next care level.

## Conclusion

The IMMENSE study population exhibited a high prevalence of PIMs and PPOs at both hospital admission and discharge. At hospital admission, PIM prevalence was 58.6% among intervention patients and 64.8% among control patients. PPO prevalence was similarly 55.3% and 55.5%. The proportion of PIMs identified at admission and resolved by discharge was higher in the intervention group (42.9%) compared to the control group (27.4%). No difference was seen for PPOs. Being allocated to the intervention group significantly reduced the risk of PIMs at discharge, while no effect was seen for PPOs. Patients living in nursing homes or home care facilities had a higher risk of PPOs at discharge. This study indicates that integrating pharmacists into the interdisciplinary healthcare team may enhance patient outcomes for older adults by reducing PIMs.

## Supplemental Material

sj-doc-1-taw-10.1177_20420986241299683 – Supplemental material for Investigating the impact of a pharmacist intervention on inappropriate prescribing practices at hospital admission and discharge in older patients: a secondary outcome analysis from a randomized controlled trial Supplemental material, sj-doc-1-taw-10.1177_20420986241299683 for Investigating the impact of a pharmacist intervention on inappropriate prescribing practices at hospital admission and discharge in older patients: a secondary outcome analysis from a randomized controlled trial by Beate Hennie Garcia, Katharina Kaino Omma, Lars Småbrekke, Jeanette Schultz Johansen, Frode Skjold and Kjell Hermann Halvorsen in Therapeutic Advances in Drug Safety

sj-docx-2-taw-10.1177_20420986241299683 – Supplemental material for Investigating the impact of a pharmacist intervention on inappropriate prescribing practices at hospital admission and discharge in older patients: a secondary outcome analysis from a randomized controlled trial Supplemental material, sj-docx-2-taw-10.1177_20420986241299683 for Investigating the impact of a pharmacist intervention on inappropriate prescribing practices at hospital admission and discharge in older patients: a secondary outcome analysis from a randomized controlled trial by Beate Hennie Garcia, Katharina Kaino Omma, Lars Småbrekke, Jeanette Schultz Johansen, Frode Skjold and Kjell Hermann Halvorsen in Therapeutic Advances in Drug Safety

sj-docx-3-taw-10.1177_20420986241299683 – Supplemental material for Investigating the impact of a pharmacist intervention on inappropriate prescribing practices at hospital admission and discharge in older patients: a secondary outcome analysis from a randomized controlled trial Supplemental material, sj-docx-3-taw-10.1177_20420986241299683 for Investigating the impact of a pharmacist intervention on inappropriate prescribing practices at hospital admission and discharge in older patients: a secondary outcome analysis from a randomized controlled trial by Beate Hennie Garcia, Katharina Kaino Omma, Lars Småbrekke, Jeanette Schultz Johansen, Frode Skjold and Kjell Hermann Halvorsen in Therapeutic Advances in Drug Safety

sj-docx-4-taw-10.1177_20420986241299683 – Supplemental material for Investigating the impact of a pharmacist intervention on inappropriate prescribing practices at hospital admission and discharge in older patients: a secondary outcome analysis from a randomized controlled trial Supplemental material, sj-docx-4-taw-10.1177_20420986241299683 for Investigating the impact of a pharmacist intervention on inappropriate prescribing practices at hospital admission and discharge in older patients: a secondary outcome analysis from a randomized controlled trial by Beate Hennie Garcia, Katharina Kaino Omma, Lars Småbrekke, Jeanette Schultz Johansen, Frode Skjold and Kjell Hermann Halvorsen in Therapeutic Advances in Drug Safety

## Declarations

**Ethics approval and consent to participate:** This research has been performed in accordance with ethical guidelines stated by the Helsinki Declaration. The Norwegian Centre for Research Data and the Norwegian Data Protection Authority granted ethical approval (Project number 41366). All participants or next of kin gave written informed consent for study participation. The IMMENSE trial was registered in clinicaltrials.gov on 28/06/2016 before enrollment started (NCT02816086).

**Consent for publication:** Not applicable.

**Availability of data and materials:** The datasets generated and analyzed during the current study are not publicly available because they contain information that can compromise research participants’ privacy/consent but are available from the corresponding author on reasonable request. Trial registration
