# Supplementary material for: Investigating the impact of a pharmacist intervention on inappropriate prescribing practices at hospital admission and discharge in older patients: a secondary outcome analysis from a randomized controlled trial
Source: Ther Adv Drug Saf. 2024 Nov 15;15:20420986241299683. doi: 10.1177/20420986241299683 (PMC11569490; doi:10.1177/20420986241299683)
Supplement: sj-docx-2-taw-10.1177_20420986241299683 – Supplemental material for Investigating the impact of a pharmacist intervention on inappropriate prescribing practices at hospital admission and discharge in older patients: a secondary outcome analysis from a randomized controlled trial [file sj-docx-2-taw-10.1177_20420986241299683.docx]

**Supplementary 1: Application guide for the STOPP/START(v2) criteria**

**Table 1: STOPP-2 criteria**

**Table 2: Anticholinergic drugs with high relative anticholinergic potency**

**Table 3: START-2 criteria**

### Table 1 Description of the STOPP-2 criteria applied, interpretation of application and reasons for exclusion (1)

| **Criterion number** | **Criterion** | **Application guide/Instructions** | **Included in assessement (Y/N)** |
| --- | --- | --- | --- |
| **Section A: Indication of medication** | | | |
| *A1* | *Any drug prescribed without an evidence-based clinical indication.* | *The dataset did not include indication for all medications use, and the criterion is consequently not applied.* | *N* |
| *A2* | *Any drug prescribed beyond the recommended duration, where treatment duration is well defined.* | *Information about length of therapy is often lacking and the criterion is consequently not applied.* | *N* |
| A3 | Any duplicate drug class prescription e.g. two concurrent NSAIDs, SSRIs, loop diuretics, ACE inhibitors, anticoagulants (optimisation of monotherapy within a single drug class should be observed prior to considering a new agent). | Not applied for different formulation types within the same medication group, such as levodopa, or for immediate-release and long-acting as in asthma treatment. | Y |
| **Section B: Cardiovascular System** | | | |
| B1 | Digoxin for heart failure with normal systolic ventricular function (no clear evidence of benefit) | The indication for digoxin is often missing. Score if the patient has diastolic heart failure without another indication, for example, use against atrial fibrillation. | Y |
| B2 | Verapamil or diltiazem with NYHA Class III or IV heart failure (may worsen heart failure). | Data on NYHA class may be missing. Score if the patient has been diagnosed with heart failure. | Y |
| B3 | Beta-blocker in combination with verapamil or diltiazem (risk of heart block). |  | Y |
| B4 | Beta blocker with bradycardia (< 50/min), type II heart block or complete heart block (risk of complete heart block, asystole). | Not scored if the patient has had a previous AV block corrected with a pacemaker. | Y |
| *B5* | *Amiodarone as first-line antiarrhythmic therapy in supraventricular tachyarrhythmias (higher risk of side-effects than beta-blockers, digoxin, verapamil or diltiazem)* | *Information about first choice of therapy did not exist. Consequently, the criterion has not been applied.* | *N* |
| *B6* | *Loop diuretic as first-line treatment for hypertension (safer, more effective alternatives available).* | *Information about first choice of therapy did not excist. Consequently, the criterion has not been applied.* | *N* |
| B7 | Loop diuretic for dependent ankle oedema without clinical, biochemical evidence or radiological evidence of heart failure, liver failure, nephrotic syndrome or renal failure (leg elevation and /or compression hosiery usually more appropriate). | The indication for loop diuretics is often missing. It is assumed to be for ankle edema if the patient does not have a diagnosis of heart failure, liver failure, nephrotic syndrome, or renal failure. | Y |
| B8 | Thiazide diuretic with current significant hypokalaemia (i.e. serum K+ < 3.0 mmol/l), hyponatraemia (i.e. serum Na+ < 130 mmol/l) hypercalcaemia (i.e. corrected serum calcium > 2.65 mmol/l) or with a history of gout (hypokalaemia, hyponatraemia, hypercalcaemia and gout can be precipitated by thiazide diuretic) |  | Y |
| *B9* | *Loop diuretic for treatment of hypertension with concurrent urinary incontinence (may exacerbate incontinence).* | *The indication for loop diuretics is mostly missing, and urinary incontinence is often not recorded as a diagnosis and is therefore also frequently missing. The criterion is consequently not applied.* | *N* |
| *B10* | *Centrally-acting antihypertensives (e.g. methyldopa, clonidine, moxonidine, rilmenidine, guanfacine), unless clear intolerance of, or lack of efficacy with, other classes of antihypertensives (centrally-active antihypertensives are generally less well tolerated by older people than younger people)* | *Information on the lack of effect from other treatments is missing in the dataset. The criterion is consequently not applied.* | *N* |
| B11 | ACE inhibitors or Angiotensin Receptor Blockers in patients with hyperkalaemia. | Hyperkalemi is defined as ≥5 mmol/l | Y |
| B12 | Aldosterone antagonists (e.g. spironolactone, eplerenone) with concurrent potassium-conserving drugs (e.g. ACEI’s, ARB’s, amiloride, triamterene) without monitoring of serum potassium (risk of dangerous hyperkalaemia i.e. > 6.0 mmol/l – serum K should be monitored regularly, i.e. at least every 6 months). | Serum potassium concentration is always measured upon hospital admission. | Y |
| B13 | Phosphodiesterase type-5 inhibitors (e.g. sildenafil, tadalafil, vardenafil) in severe heart failure characterised by hypotension i.e. systolic BP < 90 mmHg, or concurrent nitrate therapy for angina (risk of cardiovascular collapse) |  | Y |
| **Section C: Antiplatelet/Anticoagulant Drugs** | | | |
| C1 | Long-term aspirin at doses greater than 160mg per day (increased risk of bleeding, no evidence for increased efficacy). |  | Y |
| C2 | Aspirin with a past history of peptic ulcer disease without concomitant PPI (risk of recurrent peptic ulcer ). |  | Y |
| C3 | Aspirin, clopidogrel, dipyridamole, vitamin K antagonists, direct thrombin inhibitors or factor Xa inhibitors with concurrent significant bleeding risk, i.e. uncontrolled severe hypertension, bleeding diathesis, recent non-trivial spontaneous bleeding) (high risk of bleeding). | Severe hypertension is defined here as systolic blood pressure ≥180 mmHg and/or diastolic blood pressure ≥120 mmHg. | Y |
| C4 | Aspirin plus clopidogrel as secondary stroke prevention, unless the patient has a coronary stent(s) inserted in the previous 12 months or concurrent acute coronary syndrome or has a high grade symptomatic carotid arterial stenosis (no evidence of added benefit over clopidogrel monotherapy) |  | Y |
| C5 | Aspirin in combination with vitamin K antagonist, direct thrombin inhibitor or factor Xa inhibitors in patients with chronic atrial fibrillation (no added benefit from aspirin) |  | Y |
| C6 | Antiplatelet agents with vitamin K antagonist, direct thrombin inhibitor or factor Xa inhibitors in patients with stable coronary, cerebrovascular or peripheral arterial disease (No added benefit from dual therapy). | Stable coronary, cerebrovascular or peripheral arterial disease is defined as no events in the last 12 months. | Y |
| C7 | Ticlopidine in any circumstances (clopidogrel and prasugrel have similar efficacy, stronger evidence and fewer side-effects). |  | Y |
| *C8* | *Vitamin K antagonist, direct thrombin inhibitor or factor Xa inhibitors for first deep venous thrombosis without continuing provoking risk factors (e.g. thrombophilia) for > 6 months, (no proven added benefit).* | *No definition for persistent risk factors. The criterion is consequently not applied.* | *N* |
| *C9* | *Vitamin K antagonist, direct thrombin inhibitor or factor Xa inhibitors for first pulmonary embolus without continuing provoking risk factors (e.g. thrombophilia) for > 12 months (no proven added benefit).* | *No definition for persistent risk factors. The criterion is consequently not applied.* | *N* |
| C10 | NSAID and vitamin K antagonist, direct thrombin inhibitor or factor Xa inhibitors in combination (risk of major gastrointestinal bleeding). |  | Y |
| C11 | NSAID with concurrent antiplatelet agent(s) without PPI prophylaxis (increased risk of peptic ulcer disease) |  | Y |
| **Section D: Central Nervous System and Psychotropic Drugs** | | | |
| D1 | TriCyclic Antidepressants (TCAs) with dementia, narrow angle glaucoma, cardiac conduction abnormalities, prostatism, or prior history of urinary retention (risk of worsening these conditions). | Some information may be missing, for example, urinary retention. | Y |
| *D2* | *Initiation of TriCyclic Antidepressants (TCAs) as first-line antidepressant treatment (higher risk of adverse drug reactions with TCAs than with SSRIs or SNRIs).* | *No information on first-line treatment. The criterion is consequently not applied.* | *N* |
| D3 | Neuroleptics with moderate-marked antimuscarinic/anticholinergic effects (chlorpromazine, clozapine, flupenthixol, fluphenzine, pipothiazine, promazine, zuclopenthixol) with a history of prostatism or previous urinary retention (high risk of urinary retention). | If the diagnosis of prostatism is missing, the use of a 5-alpha-reductase inhibitor is used as an used as an indication. | Y |
| D4 | Selective serotonin re-uptake inhibitors (SSRI’s) with current or recent significant hyponatraemia i.e. serum Na+ < 130 mmol/l (risk of exacerbating or precipitating hyponatraemia). |  | y |
| D5 | Benzodiazepines for ≥ 4 weeks (no indication for longer treatment; risk of prolonged sedation, confusion, impaired balance, falls, road traffic accidents; all benzodiazepines should be withdrawn gradually if taken for more than 4 weeks as there is a risk of causing a benzodiazepine withdrawal syndrome if stopped abruptly). | Only regular use of benzodiazepines results in a score. It is assumed that patients with regular use of benzodiazepines without an end date have used or will use it for over 4 weeks. | Y |
| D6 | Antipsychotics (i.e. other than quetiapine or clozapine) in those with parkinsonism or Lewy Body Disease (risk of severe extra-pyramidal symptoms) |  | Y |
| D7 | Anticholinergics/antimuscarinics to treat extra-pyramidal side-effects of neuroleptic medications (risk of anticholinergic toxicity), | Information on the indication may be missing. It is assumed that patients who use biperiden together with antipsychotic medications use it to counteract side effects. Biperiden is the only medication for this purpose in Norway. | Y |
| D8 | Anticholinergics/antimuscarinics in patients with delirium or dementia (risk of exacerbation of cognitive impairment). | Anticholinergic drugs with high relative anticholinergic potency are defined in Table 2 (below) (2) | Y |
| *D9* | *Neuroleptic antipsychotic in patients with behavioural and psychological symptoms of dementia (BPSD) unless symptoms are severe and other non-pharmacological treatments have failed (increased risk of stroke).* | *Data on severity of symptoms was lacking in the dataset, also whether non-pharmacological treatments had failed. The criterion is consequently not applied.* | *N* |
| D10 | Neuroleptics as hypnotics, unless sleep disorder is due to psychosis or dementia (risk of confusion, hypotension, extra-pyramidal side effects, falls). | The indication for the medications is often missing in the dataset. Score if antipsychotics such as levomepromazine or chlorprothixene are given in a low dose in the evening and the patient does not have a known psychosis. | Y |
| D11 | Acetylcholinesterase inhibitors with a known history of persistent bradycardia (< 60 beats/min.), heart block or recurrent unexplained syncope or concurrent treatment with drugs that reduce heart rate such as beta-blockers, digoxin, diltiazem, verapamil (risk of cardiac conduction failure, syncope and injury). |  | y |
| *D12* | *Phenothiazines as first-line treatment, since safer and more efficacious alternatives exist (phenothiazines are sedative, have significant anti-muscarinic toxicity in older people, with the exception of prochlorperazine for nausea/vomiting/vertigo, chlorpromazine for relief of persistent hiccoughs and levomepromazine as an anti-emetic in palliative care ).* | *No information about first choice of therapy. The criterion is consequently not applied.* | *N* |
| D13 | Levodopa or dopamine agonists for benign essential tremor (no evidence of efficacy) |  | Y |
| D14 | First-generation antihistamines (safer, less toxic antihistamines now widely available). | Hydroxyzine (N05BB01) is defined as a first-generation antihistamine, as it is primarily used for itching in Norway. Other available options include alimemazine, promethazine, and dexchlorpheniramine. | Y |
| **Section E: Renal System. The following drugs are potentially inappropriate in older people with acute or chronic kidney disease with renal function below particular levels of eGFR (refer to summary of product characteristics datasheets and local formulary guidelines)** | | | |
| E1 | Digoxin at a long-term dose greater than 125µg/day if eGFR < 30 ml/min/1.73m2 (risk of digoxin toxicity if plasma levels not measured). | The criterion does not define long-term use. Regular use of digoxin is considered here as long-term treatment. | Y |
| E2 | Direct thrombin inhibitors (e.g. dabigatran) if eGFR < 30 ml/min/1.73m2 (risk of bleeding) |  | Y |
| E3 | Factor Xa inhibitors (e.g. rivaroxaban, apixaban) if eGFR < 15 ml/min/1.73m2 (risk of bleeding) |  | Y |
| E4 | NSAID’s if eGFR < 50 ml/min/1.73m2 (risk of deterioration in renal function). |  | Y |
| E5 | Colchicine if eGFR < 10 ml/min/1.73m2 (risk of colchicine toxicity) |  | Y |
| E6 | Metformin if eGFR < 30 ml/min/1.73m2 (risk of lactic acidosis). |  | Y |
| **Section F: Gastrointestinal System** | | | |
| F1 | Prochlorperazine or metoclopramide with Parkinsonism (risk of exacerbating Parkinsonian symptoms). |  | Y |
| F2 | PPI for uncomplicated peptic ulcer disease or erosive peptic oesophagitis at full therapeutic dosage for > 8 weeks (dose reduction or earlier discontinuation indicated). | If PPI (Proton Pump Inhibitor) is used regularly without an end date, it is considered as use over 8 weeks. Full therapeutic dose here is anything above the lowest available strength. | Y |
| *F3* | *Drugs likely to cause constipation (e.g. antimuscarinic/anticholinergic drugs, oral iron, opioids, verapamil, aluminium antacids) in patients with chronic constipation where non-constipating alternatives are available (risk of exacerbation of constipation).* | *Information about an alternative is not given. The criterion is consequently not applied.* | *N* |
| F4 | Oral elemental iron doses greater than 200 mg daily (e.g. ferrous fumarate> 600 mg/day, ferrous sulphate > 600 mg/day, ferrous gluconate> 1800 mg/day; no evidence of enhanced iron absorption above these doses). |  | Y |
| **Section G: Respiratory System** | | | |
| G1 | Theophylline as monotherapy for COPD (safer, more effective alternative; risk of adverse effects due to narrow therapeutic index). |  | Y |
| G2 | Systemic corticosteroids instead of inhaled corticosteroids for maintenance therapy in moderate-severe COPD (unnecessary exposure to long-term side-effects of systemic corticosteroids and effective inhaled therapies are available). | If COPD patients use systemic glucocorticoids and do not have inhaled glucocorticoids, this is scored regardless of whether the degree of severity is available. | Y |
| G3 | Anti-muscarinic bronchodilators (e.g. ipratropium, tiotropium) with a history of narrow angle glaucoma (may exacerbate glaucoma) or bladder outflow obstruction (may cause urinary retention). | Prostatism is defined as those with benign prostatic hyperplasia (without TUR-P).  Narrow-angle glaucoma must be specified, as most have open-angle glaucoma. | y |
| *G4* | *Non-selective beta-blocker (whether oral or topical for glaucoma) with a history of asthma requiring treatment (risk of increased bronchospasm).* | *Not applied due to a misunderstanding.* | *N* |
| *G5* | *Benzodiazepines with acute or chronic respiratory failure i.e. pO2 < 8.0 kPa ± pCO2 > 6.5 kPa (risk of exacerbation of respiratory failure).* | *Data on blood gases are missing. Criterion is consequently not applied.* | *N* |
| **Section H: Musculoskeletal System** | | | |
| H1 | Non-steroidal anti-inflammatory drug (NSAID) other than COX-2 selective agents with history of peptic ulcer disease or gastrointestinal bleeding, unless with concurrent PPI or H2 antagonist (risk of peptic ulcer relapse). |  | Y |
| H2 | NSAID with severe hypertension (risk of exacerbation of hypertension) or severe heart failure (risk of exacerbation of heart failure). | Severe hypertension is defined here as systolic blood pressure ≥180 mmHg and/or diastolic blood pressure ≥120 mmHg.  The diagnosis of heart failure results in a score. | Y |
| *H3* | *Long-term use of NSAID (>3 months) for symptom relief of osteoarthritis pain where paracetamol has not been tried (simple analgesics preferable and usually as effective for pain relief)* | *Data on previous use of paracetamol is not available. The criterion is consequently not applied.* | *N* |
| H4 | Long-term corticosteroids (>3 months) as monotherapy for rheumatoid arthrtitis (risk of systemic corticosteroid side-effects). | The indication is often missing. Score if the patient has a diagnosis of rheumatoid arthritis (without any other possible indication from the list of diagnoses) and no other medications with an RA indication. | Y |
| H5 | Corticosteroids (other than periodic intra-articular injections for mono-articular pain) for osteoarthritis (risk of systemic corticosteroid side-effects). | The indication for medications is often missing. Score if there is regular use of glucocorticoids (without any other possible indication from the list of diagnoses). | Y |
| H6 | Long-term NSAID or colchicine (>3 months) for chronic treatment of gout where there is no contraindication to a xanthine-oxidase inhibitor (e.g. allopurinol, febuxostat) (xanthine-oxidase inhibitors are first choice prophylactic drugs in gout). | Using the CAVE field in the electronic patient record to search for contraindications. Only allergic reactions are recorded in Norway as a contraindication. | Y |
| H7 | COX-2 selective NSAIDs with concurrent cardiovascular disease (increased risk of myocardial infarction and stroke) |  | Y |
| H8 | NSAID with concurrent corticosteroids without PPI prophylaxis (increased risk of peptic ulcer disease) |  | Y |
| H9 | Oral bisphosphonates in patients with a current or recent history of upper gastrointestinal disease i.e. dysphagia, oesophagitis, gastritis, duodenitis, or peptic ulcer disease, or upper gastrointestinal bleeding (risk of relapse/exacerbation of oesophagitis, oesophageal ulcer, oesophageal stricture) |  | Y |
| **Section I: Urogenital System** | | | |
| I1 | Antimuscarinic drugs with dementia, or chronic cognitive impairment (risk of increased confusion, agitation) or narrow-angle glaucoma (risk of acute exacerbation of glaucoma), or chronic prostatism (risk of urinary retention). | All medications in Norway with the ATC code G04BD (excluding mirabegron) are considered antimuscarinic agents for urinary incontinence. | Y |
| I2 | Selective alpha-1 selective alpha blockers in those with symptomatic orthostatic hypotension or micturition syncope (risk of precipitating recurrent syncope) | Patients are screened for orthostatism during admission. | Y |
| **Section J. Endocrine System** | | | |
| J1 | Sulphonylureas with a long duration of action (e.g. glibenclamide, chlorpropamide, glimepiride) with type 2 diabetes mellitus (risk of prolonged hypoglycaemia). |  | Y |
| J2 | Thiazolidenediones (e.g. rosiglitazone, pioglitazone) in patients with heart failure (risk of exacerbation of heart failure) |  | Y |
| J3 | Beta-blockers in diabetes mellitus with frequent hypoglycaemic episodes (risk of suppressing hypoglycaemic symptoms). | Information on hypoglycemic episodes may be missing. Score if the blood sugar measured is below 3.9 mmol/L or if other information about hypoglycemia is written. | y |
| J4 | Oestrogens with a history of breast cancer or venous thromboembolism (increased risk of recurrence). |  | Y |
| J5 | Oral oestrogens without progestogen in patients with intact uterus (risk of endometrial cancer). | If there is no information that a hysterectomy has been performed, the patient is considered to have an intact uterus. | Y |
| J6 | Androgens (male sex hormones) in the absence of primary or secondary hypogonadism (risk of androgen toxicity; no proven benefit outside of the hypogonadism indication). |  | Y |
| **Section K: Drugs that predictably increase the risk of falls in older people** | | | |
| K1 | Benzodiazepines (sedative, may cause reduced sensorium, impair balance). | Both regular and as-needed (pro re nata) use are scored. | Y |
| K2 | Neuroleptic drugs (may cause gait dyspraxia, Parkinsonism). | Antipsychotics are defined as all ATC codes N05A (excluding lithium). Both regular and as-needed (pro re nata) use are scored. | Y |
| K3 | Vasodilator drugs (e.g. alpha-1 receptor blockers, calcium channel blockers, long-acting nitrates, ACE inhibitors, angiotensin I receptor blockers, ) with persistent postural hypotension i.e. recurrent drop in systolic blood pressure ≥ 20mmHg (risk of syncope, falls). |  | Y |
| K4 | Hypnotic Z-drugs e.g. zopiclone, zolpidem, zaleplon (may cause protracted daytime sedation, ataxia). | Both regular and as-needed (pro re nata) use are scored. | Y |
| **Section L: Analgesic Drugs** | | | |
| *L1* | *Use of oral or transdermal strong opioids (morphine, oxycodone, fentanyl, buprenorphine, diamorphine, methadone, tramadol, pethidine, pentazocine) as first line therapy for mild pain (WHO analgesic ladder not observed).* | *No information about first choice of therapy. The criterion is consequently not applied.* | *N* |
| L2 | Use of regular (as distinct from PRN) opioids without concomitant laxative (risk of severe constipation). | Opioids are defined by ATC code N02A. As-needed (pro re nata) use is not scored.  NB! This is the same criterion as START H2. | Y |
| L3 | Long-acting opioids without short-acting opioids for break-through pain (risk of persistence of severe pain) | Opioids are defined by ATC code N02A. | Y |
| **Section N: Antimuscarinic/Anticholinergic Drug Burden** | | | |
| M1 | Concomitant use of two or more drugs with antimuscarinic/anticholinergic properties (e.g. bladder antispasmodics, intestinal antispasmodics, tricyclic antidepressants, first generation antihistamines) (risk of increased antimuscarinic/anticholinergic toxicity) | Drugs with high relative antimuscarinic/anticholinergic potency are defined in Table 2 (below). | Y |

### **Table 2:** Anticholinergic drugs with high relative anticholinergic potency as defined by Rochon PA. Drug prescribing for older adults. UpToDate <https://www.helsebiblioteket.no/> (last updated June 2020)(2)

| **Class** | **Drugs** |
| --- | --- |
| Antihistamines | H_1_ receptor antagonists, first-generation: brompheniramine, carbinoxamine, chlorpheniramine, clemastine, cyproheptadine, dimenhydrinate, diphenhydramine, doxepin, doxylamine, hydroxyzine, meclizine, triprolidine, others |
| Antiparkinson | Benztropine, trihexyphenidyl |
| Antimuscarinic, overactive bladder | Darifenacin, fesoterodine, flavoxate, oxybutynin, solifenacin, tolterodine, trospium |
| Antimuscarinic, spasmolytic | Atropine, belladonna-containing medications, clidinium-chlordiazepoxide, dicyclomine, hyoscyamine, glycopyrrolate, homatropine, methscopolamine, propantheline, scopolamine (hyoscine) |
| Antimuscarinic, inhaled bronchodilator (local effect) | Ipratropium, tiotropium |
| Antimuscarinic, ophthalmic drops (mydriatic/cycloplegic)  (local effect) | Atropine, cyclopentolate, homatropine, scopolamine |
| Gastrointestinal | Antiemetics (eg, hydroxyzine, meclizine, promethazine, scopolamine); also refer to first-generation antihistamines above |
| Muscle relaxant | Orphenadrine, tizanidine |
| Psychotropic | Antipsychotics, first-generation: chlorpromazine, fluphenazine, loxapine, methotrimeprazine (levomepromazine), thioridazine, trifluoperazine  Antipsychotics, second-generation: clozapine  Tricyclic antidepressants: amitriptyline, clomipramine, desipramine, doxepin, imipramine, nortriptyline, others |

### Table 3: Description of the START-2 criteria applied, interpretation of application and reasons for exclusion (1)

| The START criterion was not scored if it would have resulted in a STOPP criterion. For example, A7, the patient needs a beta-blocker, but has bradycardia which would have given STOPP B4. | | |  |
| --- | --- | --- | --- |
| **Criterion number** | **Criterion** | **Application guide/Instructions** | **Included in assessement (Y/N)** |
| **Section A: Cardiovascular System** | | | |
| A1 | Vitamin K antagonists or direct thrombin inhibitors or factor Xa inhibitors in the presence of chronic atrial fibrillation. | Information on whether it is paroxysmal, persistent, or chronic atrial fibrillation is not always available. Since most of the patients are over 75 years old and therefore automatically get a CHADSVASC of 2, and to simplify scoring, the diagnosis of atrial fibrillation will result in scoring. | Y |
| *A2* | *Aspirin (75 mg – 160 mg once daily) in the presence of chronic atrial fibrillation, where Vitamin K antagonists or direct thrombin inhibitors or factor Xa inhibitors are contraindicated.* | *Information on contraindications other than CAVE is not available. Not included.* | *N* |
| A3 | Antiplatelet therapy (aspirin or clopidogrel or prasugrel or ticagrelor) with a documented history of coronary, cerebral or peripheral vascular disease. | TIA is considered cerebral vascular disease, atherosclerosis alone does not. The criterion is not scored if the patient has a vitamin K antagonist, direct thrombin inhibitor, or factor Xa inhibitor and no recent cardiovascular events defined by STOPP C5 or C6. | Y |
| *A4* | *Antihypertensive therapy where systolic blood pressure consistently > 160 mmHg and/or diastolic blood pressure consistently >90 mmHg; if systolic blood pressure > 140 mmHg and/or diastolic blood pressure > 90 mmHg, if diabetic.* | *Information on persistent blood pressure is only available for patients at discharge. Since this study compares PPO at admission and discharge, this criterion is not included, as it would not provide a consistent basis for comparison.* | *N* |
| A5 | Statin therapy with a documented history of coronary, cerebral or peripheral vascular disease, unless the patient’s status is end-of-life or age is > 85 years. | Not applied to patients living permanently in nursing homes since the expected lifespan in nursing homes is relatively short. | Y |
| A6 | Angiotensin Converting Enzyme (ACE) inhibitor with systolic heart failure and/or documented coronary artery disease. | For heart failure, only applied if the patient has classified the heart failure as systolic, not diastolic. Angina pectoris is on the list for coronary disease. It is not applied if the patient has an angiotensin-II antagonist, since prescribing both is associated with more risk than benefits. | Y |
| A7 | Beta-blocker with ischaemic heart disease. | Angina pectoris is included in coronary disease. | Y |
| A8 | Appropriate beta-blocker (bisoprolol, nebivolol, metoprolol or carvedilol) with stable systolic heart failure. | Only applied if the heart failure is defined as systolic, not diastolic. | Y |
| **Section B: Respiratory System** | | | |
| B1 | Regular inhaled β2 agonist or antimuscarinic bronchodilator (e.g. ipratropium, tiotropium) for mild to moderate asthma or COPD. | Data on the severity of asthma and COPD are often missing in the dataset. The criterion is applied if the diagnosis of asthma or COPD is on the diagnosis list, and regular inhalation treatment is missing. | Y |
| B2 | Regular inhaled corticosteroid for moderate-severe asthma or COPD, where FEV1 <50% of predicted value and repeated exacerbations requiring treatment with oral corticosteroids. | Scores only if a specification on the severity is informed. | Y |
| *B3* | *Home continuous oxygen with documented chronic hypoxaemia (i.e. pO2 < 8.0 kPa or 60 mmHg or SaO2 < 89%)* | *Lacking data for blood gases, and consequently not applied.* | *N* |
| **Section C: Central Nervous System& Eyes** | | | |
| C1 | L-DOPA or a dopamine agonist in idiopathic Parkinson’s disease with functional impairment and resultant disability. | Information on functional impairment is often missing, but here it is scored for patients who have Parkinson's and assistance from the municipal health service or nursing homes without START C1 medications. | Y |
| *C2* | *Non-TCA antidepressant drug in the presence of persistent major depressive symptoms.* | *Information on the duration and severity of depression is missing. The criterion is not applied.* | *N* |
| C3 | Acetylcholinesterase inhibitor (e.g. donepezil, rivastigmine, galantamine) for mild-moderate Alzheimer’s dementia or Lewy Body dementia (rivastigmine). | Data on the severity of symptoms may be lacking. The diagnosis is considered mild or moderate if the patient does not live in a nursing home. Since most people with dementia have Alzheimer's, and to simplify the scoring, the diagnosis "dementia" is included if it is not specified that it is vascular dementia. | Y |
| C4 | Topical prostaglandin, prostamide or beta-blocker for primary open-angle glaucoma. | The diagnosis of glaucoma is considered open-angle unless otherwise specified. | Y |
| *C5* | *Selective serotonin reuptake inhibitor (or SNRI or pregabalin if SSRI contraindicated) for persistent severe anxiety that interferes with independent functioning.* | *No information on persistent severe anxiety that affects functional ability. The criterion is not applied.* | *N* |
| C6 | Dopamine agonist (ropinirole or pramipexole or rotigotine) for Restless Legs Syndrome, once iron deficiency and severe renal failure have been excluded. | Severe renal failure is considered as eGFR under 30, or renal failure grade 4. | Y |
| **Section D: Gastrointestinal System** | | | |
| *D1* | *Proton Pump Inhibitor with severe gastro-oesophageal reflux disease or peptic stricture requiring dilatation.* | *Data on the severity of gastroesophageal reflux are missing. The criterion is not applied.* | *N* |
| D2 | Fibre supplements (e.g. bran, ispaghula, methylcellulose, sterculia) for diverticulosis with a history of constipation. | Constipation history may be missing in the dataset. Score if the diagnosis is diverticulosis and the patient has been prescribed a laxative. | Y |
| **Section E: Musculoskeletal System** | | | |
| *E1* | *Disease-modifying anti-rheumatic drug (DMARD) with active, disabling rheumatoid disease.* | *No information on whether the rheumatic disease is active or disabling. The criterion is consequently not applied.* | *N* |
| E2 | Bisphosphonates and vitamin D and calcium in patients taking long-term systemic corticosteroid therapy. | All use of systemic corticosteroids is applied regardless of the dose. | y |
| E3 | Vitamin D and calcium supplement in patients with known osteoporosis and/or previous fragility fracture(s) and/or (Bone Mineral Density T-scores more than -2.5 in multiple sites). | Score if the diagnosis is osteoporosis and/or previous fracture. Information on the amount of energy is missing in the dataset, and the fracture is considered low-energy unless otherwise stated. | Y |
| E4 | Bone anti-resorptive or anabolic therapy (e.g. bisphosphonate, strontium ranelate, teriparatide, denosumab) in patients with documented osteoporosis, where no pharmacological or clinical status contraindication exists (Bone Mineral Density T-scores -> 2.5 in multiple sites) and/or previous history of fragility fracture(s). | Score if the diagnosis is osteoporosis and/or previous fracture. Information on the amount of energy is missing in the dataset, and the fracture is considered low-energy unless otherwise stated. | Y |
| E5 | Vitamin D supplement in older people who are housebound or experiencing falls or with osteopenia (Bone Mineral Density T-score is > -1.0 but < -2.5 in multiple sites). | Information on pharmacological or clinical contraindication may be difficult to find, but is excluded if not stated. | Y |
| E6 | Xanthine-oxidase inhibitors (e.g. allopurinol, febuxostat) with a history of recurrent episodes of gout. | Lacking data on who is not outdoors. Information on falls in the hospital or previously is included, but not the diagnosis "tendency to fall." Information on osteopenia is often missing. | Y |
| E7 | Folic acid supplement in patients taking methotexate. |  | Y |
| **Section F: Endocrine System** | | | |
| F1 | ACE inhibitor or Angiotensin Receptor Blocker (if intolerant of ACE inhibitor) in diabetes with evidence of renal disease i.e. dipstick proteinuria or microalbuminuria (>30mg/24 hours) with or without serum biochemical renal impairment. | Data on proteinuria is often missing, but it is scored if the patient has diabetes and an ACE inhibitor or AT-II blocker has not been prescribed and lab values have been checked for reduced kidney function. | y |
| **Section G: Urogenital System** | | | |
| G1 | Alpha-1 receptor blocker with symptomatic prostatism, where prostatectomy is not considered necessary. | Score if the patient has the diagnosis of prostatism/benign prostatic hyperplasia and there is no information that prostatectomy (TUR-P) has been performed. | Y |
| G2 | 5-alpha reductase inhibitor with symptomatic prostatism, where prostatectomy is not considered necessary. | Score if the patient has the diagnosis of prostatism/benign prostatic hyperplasia and there is no information that prostatectomy (TUR-P) has been performed. | Y |
| *G3* | *Topical vaginal oestrogen or vaginal oestrogen pessary for symptomatic atrophic vaginitis.* | *The diagnosis of atrophic vaginitis is almost always missing, and therefore the criterion is not applied.* | *N* |
| **Section H: Analgesics** | | | |
| *H1* | *High-potency opioids in moderate-severe pain, where paracetamol, NSAIDs or low-potency opioids are not appropriate to the pain severity or have been ineffective.* | *No data on pain level. The criterion is consequently not applied.* | *N* |
| H2 | Laxatives in patients receiving opioids regularly. | Opioids are defined by ATC code N02A. Pro re nata (as needed) medications are not included, only regular use. | Y |
| **Section I: Vaccines** | | | |
| *I1* | *Seasonal trivalent influenza vaccine annually* | *The dataset lacks information on vaccines. The criterion is consequently not applied.* | *N* |
| *I2* | *Pneumococcal vaccine at least once after age 65 according to national guidelines* | *The dataset lacks information on vaccines. The criterion is consequently not applied.* | *N* |

### References

1. O'Mahony D, O'Sullivan D, Byrne S, O'Connor MN, Ryan C, Gallagher P. STOPP/START criteria for potentially inappropriate prescribing in older people: version 2. Age Ageing. 2015;44(2):213-8.

2. Rochon PA. Drug prescribing for older adults: UpToDate; 2022 [updated 07.11.22. Available from: <https://www-uptodate-com.mime.uit.no/contents/drug-prescribing-for-older-adults?search=drug%20prescribing%20for%20older%20adults&source=search_result&selectedTitle=1~150&usage_type=default&display_rank=1#H6>.
