# Supplementary material for: Investigating the impact of a pharmacist intervention on inappropriate prescribing practices at hospital admission and discharge in older patients: a secondary outcome analysis from a randomized controlled trial
Source: Ther Adv Drug Saf. 2024 Nov 15;15:20420986241299683. doi: 10.1177/20420986241299683 (PMC11569490; doi:10.1177/20420986241299683)
Supplement: sj-docx-3-taw-10.1177_20420986241299683 – Supplemental material for Investigating the impact of a pharmacist intervention on inappropriate prescribing practices at hospital admission and discharge in older patients: a secondary outcome analysis from a randomized controlled trial [file sj-docx-3-taw-10.1177_20420986241299683.docx]

Supplementary 2 Directed Acyclic Graph showing model selection

Produced by <https://www.daggity.net>


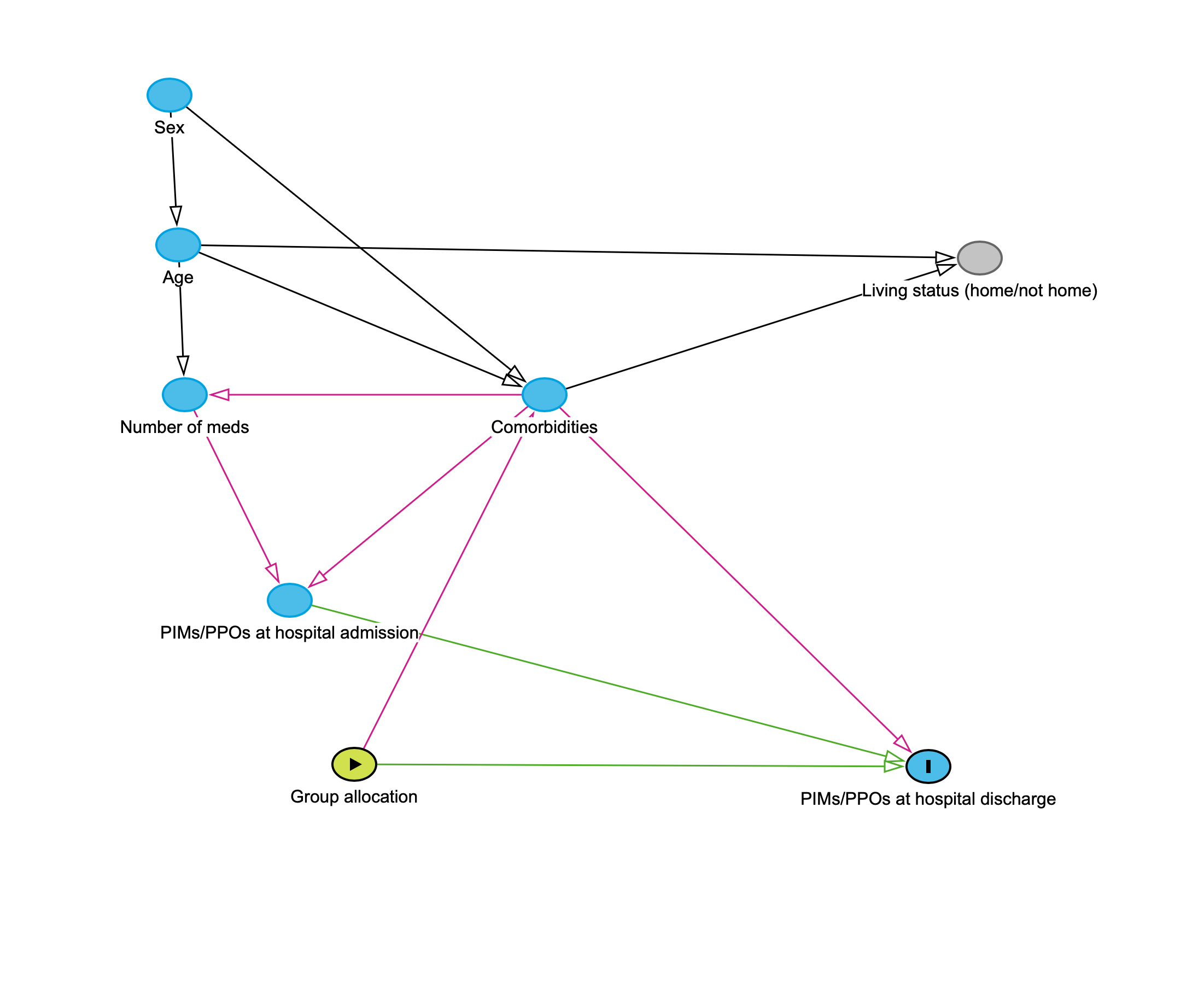


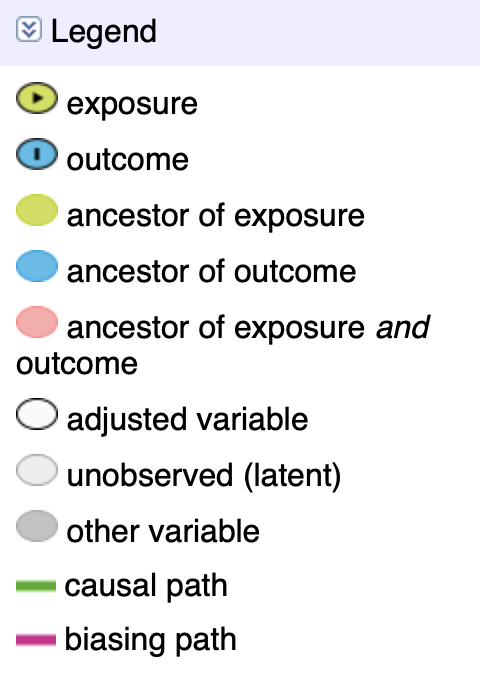


**Model code:**

dag {

"Group allocation" [exposure,pos="-0.804,0.378"]

"Living status (home/not home)" [pos="-0.292,0.012"]

"Number of meds" [pos="-0.943,0.111"]

"PIMs/PPOs at hospital admission" [pos="-0.857,0.260"]

"PIMs/PPOs at hospital discharge" [outcome,pos="-0.334,0.380"]

Age [pos="-0.948,0.003"]

Comorbidities [pos="-0.648,0.111"]

Sex [pos="-0.955,-0.106"]

"Group allocation" -> "PIMs/PPOs at hospital discharge"

"Group allocation" -> Comorbidities

"Number of meds" -> "PIMs/PPOs at hospital admission"

"PIMs/PPOs at hospital admission" -> "PIMs/PPOs at hospital discharge"

Age -> "Living status (home/not home)"

Age -> "Number of meds"

Age -> Comorbidities

Comorbidities -> "Living status (home/not home)"

Comorbidities -> "Number of meds"

Comorbidities -> "PIMs/PPOs at hospital admission"

Comorbidities -> "PIMs/PPOs at hospital discharge"

Sex -> Age

Sex -> Comorbidities

}
