# Supplementary material for: Investigating the impact of a pharmacist intervention on inappropriate prescribing practices at hospital admission and discharge in older patients: a secondary outcome analysis from a randomized controlled trial
Source: Ther Adv Drug Saf. 2024 Nov 15;15:20420986241299683. doi: 10.1177/20420986241299683 (PMC11569490; doi:10.1177/20420986241299683)
Supplement: sj-docx-4-taw-10.1177_20420986241299683 – Supplemental material for Investigating the impact of a pharmacist intervention on inappropriate prescribing practices at hospital admission and discharge in older patients: a secondary outcome analysis from a randomized controlled trial [file sj-docx-4-taw-10.1177_20420986241299683.docx]

**Supplementary 3**

**Overview of STOPP(v2) and START(v2) criterion applications in intervention group (n=244) and control group (n=236) patients**

## Table 1: STOPP criteria applications

| Criterion number | Criterion description | Intervention group | | | | Control group | | | |
| --- | --- | --- | --- | --- | --- | --- | --- | --- | --- |
|  |  | **Admission** | | **Discharge** | | **Admission** | | **Discharge** | |
|  |  | **n** | **%** | **N** | **%** | **n** | **%** | **N** | **%** |
| A3 | Any duplicate drug class prescription e.g. two concurrent NSAIDs, SSRIs, loop diuretics, ACE inhibitors, anticoagulants | 15 | 6,1 | 12 | 4,9 | 16 | 6,8 | 24 | 10,2 |
| B2 | Verapamil or diltiazem with NYHA Class III or IV heart failure (may worsen heart failure). | 1 | 0,4 | 0 | 0 | 0 | 0 | 0 | 0 |
| B3 | Beta-blocker in combination with verapamil or diltiazem (risk of heart block). | 1 | 0,4 | 1 | 0,4 | 2 | 0,8 | 1 | 0,4 |
| B4 | Beta blocker with bradycardia (< 50/min), type II heart block or complete heart block (risk of complete heart block, asystole). | 1 | 0,4 | 1 | 0,4 | 0 | 0 | 0 | 0 |
| B7 | Loop diuretic for dependent ankle oedema without clinical, biochemical evidence or radiological evidence of heart failure, liver failure, nephrotic syndrome or renal failure (leg elevation and /or compression hosiery usually more appropriate). | 20 | 8,2 | 12 | 4,9 | 20 | 8,4 | 11 | 4,7 |
| B8 | Thiazide diuretic with current significant hypokalaemia (i.e. serum K+ < 3.0 mmol/l), hyponatraemia (i.e. serum Na+ < 130 mmol/l) hypercalcaemia (i.e. corrected serum calcium > 2.65 mmol/l) or with a history of gout (hypokalaemia, hyponatraemia, hypercalcaemia and gout can be precipitated by thiazide diuretic) | 3 | 1,2 | 1 | 0,4 | 2 | 0,8 | 0 | 0 |
| B11 | ACE inhibitors or Angiotensin Receptor Blockers in patients with hyperkalaemia. | 7 | 2,9 | 2 | 0,8 | 5 | 2,1 | 4 | 1,7 |
| C2 | Aspirin with a past history of peptic ulcer disease without concomitant PPI (risk of recurrent peptic ulcer ). | 2 | 0,8 | 2 | 0,8 | 1 | 0,4 | 1 | 0,4 |
| C3 | Aspirin, clopidogrel, dipyridamole, vitamin K antagonists, direct thrombin inhibitors or factor Xa inhibitors with concurrent significant bleeding risk, i.e. uncontrolled severe hypertension, bleeding diathesis, recent non-trivial spontaneous bleeding) (high risk of bleeding). | 6 | 2,5 | 3 | 1,2 | 2 | 0,8 | 6 | 2,5 |
| C4 | Aspirin plus clopidogrel as secondary stroke prevention, unless the patient has a coronary stent(s) inserted in the previous 12 months or concurrent acute coronary syndrome or has a high grade symptomatic carotid arterial stenosis (no evidence of added benefit over clopidogrel monotherapy) | 0 | 0 | 1 | 0,4 | 0 | 0 | 0 | 0 |
| C5 | Aspirin in combination with vitamin K antagonist, direct thrombin inhibitor or factor Xa inhibitors in patients with chronic atrial fibrillation (no added benefit from aspirin) | 3 | 1,2 | 0 | 0 | 8 | 3,4 | 6 | 2,5 |
| C10 | NSAID and vitamin K antagonist, direct thrombin inhibitor or factor Xa inhibitors in combination (risk of major gastrointestinal bleeding). | 1 | 0,4 | 2 | 0,8 | 4 | 1,7 | 4 | 1,7 |
| D1 | TriCyclic Antidepressants (TCAs) with dementia, narrow angle glaucoma, cardiac conduction abnormalities, prostatism, or prior history of urinary retention (risk of worsening these conditions). | 0 | 0 | 1 | 0,4 | 2 | 0,8 | 4 | 1,7 |
| D4 | Selective serotonin re-uptake inhibitors (SSRI’s) with current or recent significant hyponatraemia i.e. serum Na+ < 130 mmol/l (risk of exacerbating or precipitating hyponatraemia). | 1 | 0,4 | 0 | 0 | 1 | 0,4 | 0 | 0 |
| D5 | Benzodiazepines for ≥ 4 weeks (no indication for longer treatment; risk of prolonged sedation, confusion, impaired balance, falls, road traffic accidents; all benzodiazepines should be withdrawn gradually if taken for more than 4 weeks as there is a risk of causing a benzodiazepine withdrawal syndrome if stopped abruptly). | 9 | 3,7 | 9 | 3,7 | 15 | 6,4 | 13 | 5,5 |
| D6 | Antipsychotics (i.e. other than quetiapine or clozapine) in those with parkinsonism or Lewy Body Disease (risk of severe extra-pyramidal symptoms) | 0 | 0 | 0 | 0 | 2 | 0,8 | 2 | 0,8 |
| D7 | Anticholinergics/antimuscarinics to treat extra-pyramidal side-effects of neuroleptic medications (risk of anticholinergic toxicity), | 1 | 0,4 | 1 | 0,4 | 1 | 0,4 | 1 | 0,4 |
| D8 | Anticholinergics/antimuscarinics in patients with delirium or dementia (risk of exacerbation of cognitive impairment). | 5 | 2 | 6 | 2,5 | 6 | 2,5 | 11 | 4,7 |
| D10 | Neuroleptics as hypnotics, unless sleep disorder is due to psychosis or dementia (risk of confusion, hypotension, extra-pyramidal side effects, falls). | 3 | 1,2 | 3 | 1,2 | 1 | 0,4 | 1 | 0,4 |
| D11 | Acetylcholinesterase inhibitors with a known history of persistent bradycardia (< 60 beats/min.), heart block or recurrent unexplained syncope or concurrent treatment with drugs that reduce heart rate such as beta-blockers, digoxin, diltiazem, verapamil (risk of cardiac conduction failure, syncope and injury). | 3 | 1,2 | 2 | 0,8 | 2 | 0,8 | 1 | 0,4 |
| D13 | Levodopa or dopamine agonists for benign essential tremor (no evidence of efficacy) | 1 | 0,4 | 1 | 0,4 | 0 | 0 | 0 | 0 |
| D14 | First-generation antihistamines (safer, less toxic antihistamines now widely available). | 7 | 2,9 | 4 | 1,6 | 7 | 3 | 7 | 3 |
| E3 | Factor Xa inhibitors (e.g. rivaroxaban, apixaban) if eGFR < 15 ml/min/1.73m2 (risk of bleeding) | 1 | 0,4 | 0 | 0 | 0 | 0 | 0 | 0 |
| E4 | NSAID’s if eGFR < 50 ml/min/1.73m2 (risk of deterioration in renal function). | 3 | 1,2 | 4 | 1,6 | 3 | 1,3 | 2 | 0,8 |
| E6 | Metformin if eGFR < 30 ml/min/1.73m2 (risk of lactic acidosis). | 3 | 1,2 | 0 | 0 | 4 | 1,7 | 0 | 0 |
| F1 | Prochlorperazine or metoclopramide with Parkinsonism (risk of exacerbating Parkinsonian symptoms). | 0 | 0 | 0 | 0 | 1 | 0,4 | 1 | 0,4 |
| F2 | PPI for uncomplicated peptic ulcer disease or erosive peptic oesophagitis at full therapeutic dosage for > 8 weeks (dose reduction or earlier discontinuation indicated). | 16 | 6,6 | 13 | 5,3 | 30 | 12,7 | 25 | 10,6 |
| G2 | Theophylline as monotherapy for COPD (safer, more effective alternative; risk of adverse effects due to narrow therapeutic index). | 0 | 0 | 0 | 0 | 1 | 0,4 | 1 | 0,4 |
| G3 | Systemic corticosteroids instead of inhaled corticosteroids for maintenance therapy in moderate-severe COPD (unnecessary exposure to long-term side-effects of systemic corticosteroids and effective inhaled therapies are available). | 3 | 1,2 | 2 | 0,8 | 6 | 2,5 | 6 | 2,5 |
| H1 | Non-steroidal anti-inflammatory drug (NSAID) other than COX-2 selective agents with history of peptic ulcer disease or gastrointestinal bleeding, unless with concurrent PPI or H2 antagonist (risk of peptic ulcer relapse). | 0 | 0 | 0 | 0 | 2 | 0,8 | 1 | 0,4 |
| H2 | NSAID with severe hypertension (risk of exacerbation of hypertension) or severe heart failure (risk of exacerbation of heart failure). | 1 | 0,4 | 4 | 1,6 | 2 | 0,8 | 4 | 1,7 |
| H4 | Long-term corticosteroids (>3 months) as monotherapy for rheumatoid arthrtitis (risk of systemic corticosteroid side-effects). | 0 | 0 | 0 | 0 | 1 | 0,4 | 1 | 0,4 |
| H6 | Long-term NSAID or colchicine (>3 months) for chronic treatment of gout where there is no contraindication to a xanthine-oxidase inhibitor (e.g. allopurinol, febuxostat) (xanthine-oxidase inhibitors are first choice prophylactic drugs in gout). | 1 | 0,4 | 2 | 0,8 | 2 | 0,8 | 3 | 1,7 |
| H8 | NSAID with concurrent corticosteroids without PPI prophylaxis (increased risk of peptic ulcer disease) | 1 | 0,4 | 0 | 0 | 2 | 0,8 | 1 | 0,4 |
| I1 | Antimuscarinic drugs with dementia, or chronic cognitive impairment (risk of increased confusion, agitation) or narrow-angle glaucoma (risk of acute exacerbation of glaucoma), or chronic prostatism (risk of urinary retention). | 1 | 0,4 | 1 | 0,4 | 2 | 0,8 | 3 | 1,3 |
| I2 | Selective alpha-1 selective alpha blockers in those with symptomatic orthostatic hypotension or micturition syncope (risk of precipitating recurrent syncope) | 2 | 0,8 | 4 | 1,6 | 3 | 1,3 | 4 | 1,7 |
| J1 | Sulphonylureas with a long duration of action (e.g. glibenclamide, chlorpropamide, glimepiride) with type 2 diabetes mellitus (risk of prolonged hypoglycaemia). | 3 | 1,2 | 1 | 0,4 | 5 | 2,1 | 4 | 1,7 |
| J3 | Beta-blockers in diabetes mellitus with frequent hypoglycaemic episodes (risk of suppressing hypoglycaemic symptoms). | 0 | 0 | 0 | 0 | 1 | 0,4 | 1 | 0,4 |
| J5 | Oral oestrogens without progestogen in patients with intact uterus (risk of endometrial cancer). | 6 | 2,5 | 5 | 2 | 2 | 0,8 | 1 | 0,4 |
| J6 | Androgens (male sex hormones) in the absence of primary or secondary hypogonadism (risk of androgen toxicity; no proven benefit outside of the hypogonadism indication). | 0 | 0 | 1 | 0,4 | 0 | 0 | 0 | 0 |
| K1 | Benzodiazepines (sedative, may cause reduced sensorium, impair balance). | 42 | 17,2 | 37 | 15,2 | 43 | 18,2 | 49 | 20,8 |
| K2 | Neuroleptic drugs (may cause gait dyspraxia, Parkinsonism). | 13 | 5,3 | 14 | 5,7 | 18 | 7,6 | 23 | 9,7 |
| K3 | Vasodilator drugs (e.g. alpha-1 receptor blockers, calcium channel blockers, long-acting nitrates, ACE inhibitors, angiotensin I receptor blockers, ) with persistent postural hypotension i.e. recurrent drop in systolic blood pressure ≥ 20mmHg (risk of syncope, falls). | 14 | 5,7 | 11 | 4,5 | 14 | 5,9 | 15 | 6,4 |
| K4 | Hypnotic Z-drugs e.g. zopiclone, zolpidem, zaleplon (may cause protracted daytime sedation, ataxia). | 48 | 19,7 | 59 | 24,2 | 52 | 22 | 56 | 23,7 |
| L2 | Use of regular (as distinct from PRN) opioids without concomitant laxative (risk of severe constipation). | 18 | 7,4 | 12 | 4,9 | 21 | 8,9 | 13 | 6,4 |
| L3 | Long-acting opioids without short-acting opioids for break-through pain (risk of persistence of severe pain) | 13 | 5,3 | 12 | 4,9 | 13 | 5,5 | 13 | 6,4 |
| M1 | Concomitant use of two or more drugs with antimuscarinic/anticholinergic properties (e.g. bladder antispasmodics, intestinal antispasmodics, tricyclic antidepressants, first generation antihistamines) (risk of increased antimuscarinic/anticholinergic toxicity) | 10 | 4,1 | 6 | 2,5 | 7 | 3 | 12 | 5,1 |
| *NSAID: non-steroidal anti-inflammatory drugs, ACE inhibitor; angiotensin-converting enzyme inhibitor, ASA; acetylsalicylic acid, TCA; tricyclic antidepressants, ICS; inhaled corticosteroids, COPD; chronic obstructive pulmonary disease, RA; rheumatoid arthritis, SSRI; selective serotonin reuptake inhibitor, COX; Cyclooxygenase, GI tract; gastrointestinal tract, DVT; deep vein thrombosis* | | | | | | | | | |

## Table 2: START criteria applications

| **Criterion** | **Description of criterion** | **Intervention group** | | | | **Control group** | | | |
| --- | --- | --- | --- | --- | --- | --- | --- | --- | --- |
|  |  | **Admission** | | **Discharge** | | **Inn** | | **Ut** | |
|  |  | **n** | **%** | **n** | **%** | **n** | **%** | **N** | **%** |
| **A1** | Vitamin K antagonists or direct thrombin inhibitors or factor Xa inhibitors in the presence of chronic atrial fibrillation. | 4 | 1,6 | 7 | 2,9 | 6 | 2,5 | 7 | 3 |
| **A3** | Antiplatelet therapy (aspirin or clopidogrel or prasugrel or ticagrelor) with a documented history of coronary, cerebral or peripheral vascular disease. | 7 | 2,9 | 10 | 4,1 | 11 | 4,7 | 7 | 3 |
| **A5** | Statin therapy with a documented history of coronary, cerebral or peripheral vascular disease, unless the patient’s status is end-of-life or age is > 85 years. | 12 | 4,9 | 12 | 4,9 | 19 | 8,1 | 21 | 8,9 |
| **A6** | Angiotensin Converting Enzyme (ACE) inhibitor with systolic heart failure and/or documented coronary artery disease. | 16 | 6,6 | 22 | 9 | 22 | 9,3 | 24 | 10,2 |
| **A7** | Beta-blocker with ischaemic heart disease. | 10 | 4,1 | 14 | 5,7 | 17 | 7,2 | 19 | 8,1 |
| **A8** | Appropriate beta-blocker (bisoprolol, nebivolol, metoprolol or carvedilol) with stable systolic heart failure. | 0 | 0 | 0 | 0 | 0 | 0 | 0 | 0 |
| **B1** | Regular inhaled β2 agonist or antimuscarinic bronchodilator (e.g. ipratropium, tiotropium) for mild to moderate asthma or COPD. | 15 | 6,1 | 16 | 6,6 | 12 | 5,1 | 12 | 5,1 |
| **B2** | Regular inhaled corticosteroid for moderate-severe asthma or COPD, where FEV1 <50% of predicted value and repeated exacerbations requiring treatment with oral corticosteroids. | 1 | 0,4 | 1 | 0,4 | 1 | 0,4 | 1 | 0,4 |
| **C1** | L-DOPA or a dopamine agonist in idiopathic Parkinson’s disease with functional impairment and resultant disability. | 1 | 0,4 | 2 | 0,8 | 1 | 0,4 | 1 | 0,4 |
| **C3** | Acetylcholinesterase inhibitor (e.g. donepezil, rivastigmine, galantamine) for mild-moderate Alzheimer’s dementia or Lewy Body dementia (rivastigmine). | 10 | 4,1 | 11 | 4,5 | 6 | 2,5 | 16 | 6,8 |
| **C4** | Topical prostaglandin, prostamide or beta-blocker for primary open-angle glaucoma. | 0 | 0 | 0 | 0 | 2 | 0,8 | 2 | 0,8 |
| **C6** | Dopamine agonist (ropinirole or pramipexole or rotigotine) for Restless Legs Syndrome, once iron deficiency and severe renal failure have been excluded. | 0 | 0 | 0 | 0 | 0 | 0 | 0 | 0 |
| **D2** | Fibre supplements (e.g. bran, ispaghula, methylcellulose, sterculia) for diverticulosis with a history of constipation. | 1 | 0,4 | 10 | 4,1 | 2 | 0,8 | 6 | 2,5 |
| **E2** | Bisphosphonates and vitamin D and calcium in patients taking long-term systemic corticosteroid therapy. | 14 | 5,7 | 11 | 4,5 | 18 | 7,6 | 17 | 7,2 |
| **E3** | Vitamin D and calcium supplement in patients with known osteoporosis and/or previous fragility fracture(s) and/or (Bone Mineral Density T-scores more than -2.5 in multiple sites). | 40 | 16,4 | 37 | 15,2 | 41 | 17,4 | 44 | 18,6 |
| **E4** | Bone anti-resorptive or anabolic therapy (e.g. bisphosphonate, strontium ranelate, teriparatide, denosumab) in patients with documented osteoporosis, where no pharmacological or clinical status contraindication exists (Bone Mineral Density T-scores -> 2.5 in multiple sites) and/or previous history of fragility fracture(s). | 57 | 23,4 | 56 | 23 | 47 | 19,9 | 47 | 19,9 |
| **E5** | Vitamin D supplement in older people who are housebound or experiencing falls or with osteopenia (Bone Mineral Density T-score is > -1.0 but < -2.5 in multiple sites). | 8 | 3,3 | 11 | 4,5 | 1 | 0,4 | 3 | 1,3 |
| **E6** | Xanthine-oxidase inhibitors (e.g. allopurinol, febuxostat) with a history of recurrent episodes of gout. | 7 | 2,9 | 5 | 2 | 5 | 2,1 | 6 | 2,5 |
| **E7** | Folic acid supplement in patients taking methotexate. | 0 | 0 | 0 | 0 | 0 | 0 | 0 | 0 |
| **F1** | ACE inhibitor or Angiotensin Receptor Blocker (if intolerant of ACE inhibitor) in diabetes with evidence of renal disease i.e. dipstick proteinuria or microalbuminuria (>30mg/24 hours) with or without serum biochemical renal impairment. | 6 | 2,5 | 8 | 3,3 | 7 | 3 | 5 | 2,1 |
| **G1** | Alpha-1 receptor blocker with symptomatic prostatism, where prostatectomy is not considered necessary. | 8 | 3,3 | 10 | 4,1 | 7 | 3 | 6 | 2,5 |
| **G2** | 5-alpha reductase inhibitor with symptomatic prostatism, where prostatectomy is not considered necessary. | 7 | 2,9 | 8 | 3,3 | 10 | 4,2 | 10 | 4,2 |
| **H2** | Laxatives in patients receiving opioids regularly. | 18 | 7,4 | 12 | 4,9 | 21 | 8,9 | 13 | 5,5 |
| *ACE inhibitor; angiotensin-converting enzyme inhibitor, COPD; chronic obstructive pulmonary disease, ICS; inhaled corticosteroids* | | | | | | | | | |
